# Flow Chemistry as an Enabling Technology for Process-Intensified Amination Reactions: A Decadal Review

**DOI:** 10.3390/molecules31071151

**Published:** 2026-03-31

**Authors:** Feng Zhou, Yijun Zhou, Pan Wang, Yanxing Li, Jin Li, Haiqing Xu, Chuansong Duanmu

**Affiliations:** National & Local Joint Engineering Research Center for Deep Utilization Technology of Rock-Salt Resource, Faculty of Chemical Engineering, Huaiyin Institute of Technology, Huai’an 223003, China

**Keywords:** amination, microreactor, flow chemistry, process intensification

## Abstract

Amines are indispensable structural motifs in pharmaceuticals, agrochemicals, functional materials, and beyond, driving continuous demand for efficient synthetic methods. While established strategies like cross-coupling and reductive amination are prevalent, traditional batch processes often suffer from limitations in mixing, heat/mass transfer, safety, and scalability. Flow chemistry emerges as a powerful process intensification technology, offering enhanced transport properties, precise parameter control, and improved safety profiles, thereby presenting a highly efficient approach for amine synthesis. This review systematically summarizes representative advances in flow chemistry for amination reactions from 2015 onward. It encompasses a broad range of enabling scenarios (e.g., heterogeneous, thermally activated, and enzymatic amination, among others), analyzed through the lens of process intensification. This review also examines the development of novel continuous-flow amination processes and the study of reaction kinetics leveraging flow chemistry. By providing a consolidated reference on the field’s evolution over the past decade, this review aims to guide researchers toward developing more efficient, sustainable, and scalable flow-based amination processes.

## 1. Introduction

Amines are an important class of derivatives of ammonia, in which one or more hydrogen atoms are replaced by organic groups [1]. As indispensable bulk and fine chemicals, amines are key molecular building blocks that drive a vast array of industries, such as the production of pharmaceuticals, agrochemicals, and high-performance materials [2,3,4]. Given the significant application value of amines, the development of efficient and convenient synthetic methods has been an important research focus in modern synthetic chemistry. Currently, prevalent strategies for constructing C-N bond include methods such as metal-catalyzed C-N cross-coupling, reductive amination, and electrophilic amination [2,5]. Despite ongoing improvements in these established strategies, the exploration of novel and efficient amination pathways to rapidly access structurally diverse C-N bonds remains an important research direction in this field [6,7,8,9,10,11,12]. At the same time, intensifying amination reactions from a process engineering perspective has garnered increasing attention. Traditional amination processes are predominantly carried out in batch reactors, which face challenges such as inadequate mixing, limited mass and heat transfer, safety concerns, and insufficient batch-to-batch reproducibility. Flow chemistry, as a representative process intensification technology, offers a promising solution for amination reactions due to its efficient mixing and mass/heat transfer, precise control over temperature and residence time, enhanced process safety, and improved scalability [13]. It has already demonstrated significant advantages across various reaction types and provides an important pathway toward more efficient and sustainable amine synthesis [13,14,15,16].

Researchers have conducted review discussions on the application of flow chemistry technology in amination reactions [7,17,18]. Bukhtiyarova et al. [17] systematically reviewed the research progress on the reductive amination of levulinic acid and its derivatives to synthesize pyrrolidones via heterogeneous catalysis. Currently, studies on this reaction system remain predominantly based on batch reactors, which commonly suffer from limitations such as low mass and heat transfer efficiency, cumbersome post-processing, and challenges in scale-up. In contrast, continuous-flow systems, with their superior transport properties, precise parameter controllability, and online monitoring capabilities, provide an effective approach for process intensification; rapid condition optimization; and safe, green synthesis. Although research on this system under flow conditions is still in its early stages, and catalytic performance in batch mode remains superior, flow chemistry demonstrates significant potential in enhancing mixing, simplifying downstream processing, inhibiting active site deactivation, and facilitating process scale-up. Zhang et al. [18] systematically reviewed recent advances in catalytic hydrogenation-mediated reductive amination under continuous-flow conditions. Their work comprehensively examines the influence of catalysts, solvents, temperature, additives, and substrate properties on the reaction and summarizes flow chemistry systems employing ammonia, amines, and nitro compounds as nitrogen sources. It also provides an in-depth analysis of how precise temperature control, enhanced mass transfer, and regulated residence time in continuous-flow reactors contribute to improved reaction selectivity. Furthermore, the review outlines future challenges and prospects in catalyst design, process development, and industrial application, offering valuable insights for the further development of flow-based strategies in amine synthesis. Saunders and colleagues [7] have summarized key challenges in applying flow chemistry to Pd-catalyzed amination reactions. While such reactions could theoretically benefit from flow chemistry technology, practical implementation faces obstacles, including high Pd loadings, extended reaction times, and the routine use of organic solvents. A particularly critical issue in flow-based C-N bond formation is the limited solubility of starting materials, products, and inorganic salt by-products in organic solvents, which can cause clogging and eventual reactor failure. Since plug flow operations typically occur in coil or packed-bed reactors, maintaining steady-state, precipitation-free conditions is essential, yet especially difficult for Pd-catalyzed amination in continuous flow. The authors also reviewed several flow chemistry strategies developed to address these challenges, including aminations in aqueous plug flow systems, the use of ionic liquid-forming organic bases, biphasic amination processes, and methods for catalyst recycling.

While several reviews have explored the application of flow chemistry in amination reactions, they are primarily confined to specific subfields. This review systematically summarizes representative advances in flow chemistry for amination reactions since 2015 from a process intensification perspective. It encompasses a wide array of methods within the key enabling scenarios of flow chemistry, such as heterogeneous amination, thermally activated amination, and enzymatic amination. Thereby, it offers a comprehensive overview of the field’s progress over the past decade and serves as an integrated reference for researchers.

## 2. Heterogeneous Amination Processes

Heterogeneous amination processes involve the combination of two or more immiscible phases, such as gas–liquid, solid–liquid, or gas–liquid–solid systems. In such reactions, interphase mass transfer is often the rate-determining step. Therefore, reactor design is crucial for achieving efficient mass transfer between phases, with a key focus on maximizing the interfacial contact area to accelerate transfer processes. The unique characteristic of continuous-flow reactors gives them a notable advantage in multiphase reactions, resulting in superior mass transfer efficiency compared to traditional batch reactors.

### 2.1. Gas–Liquid Processes

In gas–liquid amination processes, conventional batch methods often suffer from limited mass transfer due to poor gas solubility and restricted interfacial contact area, leading to slow reaction rates and the need for stoichiometric excess of gaseous reagents. Continuous-flow systems can effectively enhance mass transfer efficiency and significantly accelerate mass transfer-limited reactions due to their high surface-area-to-volume ratio. Furthermore, flow systems can operate under elevated pressure, increasing gas solubility in the reaction medium. Integrated devices such as mass flow controllers enable precise, reproducible dosing of gaseous reactants, improving both safety and stoichiometric control. Overall, continuous-flow processes not only intensify interfacial mixing and gas dissolution but also provide accurate chemical control, resulting in faster reactions, better scalability, and safer operation.

**Figure 1:**
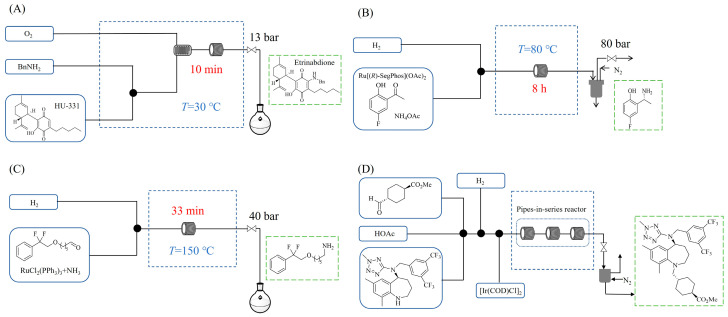
Continuous-flow processes for gas–liquid amination reactions. (**A**) Segmented flow oxidative amination. (**B**) Ru-catalyzed asymmetric reductive amination. (**C**) Ru-catalyzed reductive amination. (**D**) Ir-catalyzed reductive amination.

Zielke et al. [19] developed a two-step continuous-flow process for synthesizing Etrinabdione from cannabidiol, with the second step involving a gas–liquid continuous-flow oxidative amination reaction. This step converts HU-331 to Etrinabdione using benzylamine as the nucleophile and molecular oxygen as the oxidant, operating under segmented flow conditions to enhance mass transfer and safety. A high isolated yield of 99% for Etrinabdione from HU-331 was achieved by optimizing key parameters for the second step (Figure 1A), including equivalents of benzylamine (10 eq.), oxygen stoichiometry (1.5 eq.), residence time (10 min), and temperature (30 °C). The telescoped two-step process, incorporating a gravity-based continuous separation, afforded Etrinabdione in 98% yield and achieved a 97% reduction in process mass intensity (PMI) compared to the batch protocol. Xu et al. [20] presents a highly enantioselective synthesis of the chiral primary amine (*R*)-2-(1-aminoethyl)-4-fluorophenol, a key intermediate for the tyrosine kinase inhibitor repotrectinib, via Ru-catalyzed asymmetric reductive amination. Starting from ketone 1-(5-fluoro-2-hydroxyphenyl)ethan-1-one, the reaction utilizes ammonium acetate as the ammonia source and hydrogen gas as the reducing agent. To address scalability limitations observed in batch reactions, notably significant alcohol by-product formation during gram-scale batch experiments, the authors transitioned the process to a continuous-flow system to enhance both safety and efficiency. Following systematic optimization, the conditions for the continuous-flow process were established as a substrate concentration of 0.0875 M in methanol, 0.66 mol% Ru[(*R*)-SegPhos](OAc)_2_ catalyst, pressure 80 atm, residence time 8 h, and reaction temperature 80 °C, enabling a successful scale-up to 21.6 g of substrate to afford the target amine in 82% isolated yield with 99% ee (Figure 1B). García-Lacuna et al. [21] demonstrates a multi-step synthesis of the lipophilic amine tail of abediterol, wherein a high-pressure reductive amination serves as a key gas–liquid transformation. The authors developed a telescoped continuous-flow sequence comprising a phase-transfer-catalyzed O-alkylation and a rhodium-catalyzed hydroformylation, followed by a ruthenium-catalyzed reductive amination that required a solvent switch prior to its execution. Application of the optimized reaction conditions (RuCl_2_(PPh_3_)_3_ catalyst, 7 N NH_3_ in MeOH, 40 bar H_2_, 150 °C) to the crude telescoped O-alkylation/hydroformylation output yielded the primary amine in 50% isolated yield (Figure 1C). The three-step continuous-flow sequence afforded the target amine in a combined yield of 38% after acid/base extraction, demonstrating a significant improvement over the 26% yield reported for an alternative batch synthesis. May et al. [22] presents an example of scaling a homogeneous iridium-catalyzed reductive amination from laboratory research to GMP production for the synthesis of evacetrapib’s penultimate intermediate. The authors developed a continuous-flow process (Figure 1D) to address the limitations of previous batch routes that employed stoichiometric sodium triacetoxyborohydride or heterogeneous Pt/C catalysis, which posed challenges in safety, cost, and scalability. The optimized continuous-flow system utilized [Ir(COD)Cl]_2_ as the catalyst with tetrabutylammonium iodide as a critical additive to suppress iridium plating and enhance catalyst stability. A custom-designed “pipes-in-series” plug-flow reactor was employed to ensure efficient gas–liquid mass transfer and operational safety. During a 24-day GMP campaign, the process demonstrated remarkable stability, producing over 2 metric tons of the tertiary amine intermediate in 95% yield after workup and crystallization.

### 2.2. Solid–Liquid Processes

Solid–liquid amination, as a key category of heterogeneous amination reactions, has achieved significant process intensification through flow chemistry technology. Such reactions are typically conducted in the continuous-flow packed-bed reactor, a configuration that immobilizes catalyst particles, thereby enhancing mass transfer and simplifying downstream separation. Flow chemistry also enables assessment of solid catalyst stability under controlled reaction conditions, providing more reliable deactivation profiles and longer-term performance data compared to batch methods. This section examines representative examples of solid–liquid amination processes under flow mode. A summary of typical examples for the solid–liquid amination processes under continuous-flow mode is given in Table 1, and the information about process parameter is also listed [23,24,25,26,27,28,29,30,31,32,33,34,35,36].

Solid–liquid amination processes typically rely on a catalyst, with the solid phase often being a metal catalyst. Earth-abundant metal catalysts, such as iron, nickel, and copper, offer cost-effective and sustainable alternatives for continuous-flow amination processes. Donato et al. [23] developed Cu(OAc)_2_·H_2_O as the catalyst with K_3_PO_4_ for the copper-mediated C-H activation. The flow amination process enables efficient synthesis of Ethyl 5-(butylamino)-1-(3-methoxypyridin-2-yl)-1H-1,2,3-triazole-4-carboxylate through Cu-catalyzed C-H activation in a packed-bed reactor with significantly enhanced productivity (69×) and space-time yield (23×) compared to batch methods (Table 1, entry 1). Nishizawa et al. [24] established a highly efficient continuous-flow aminolysis of epoxides catalyzed by a titania-zirconia supported molybdenum oxide catalyst, enabling the conversion of various aromatic amines into corresponding β-amino alcohols with high yield and productivity (e.g., ~80% yield for 1-chloro-3-(phenylamino)propan-2-ol (Table 1, entry 2)). Ji et al. [25] developed a strongly Lewis-acidic metal–organic framework (ZrOTf-BTC) by postsynthetic triflation of the Zr_6_ nodes of MOF-808, yielding a solid catalyst with acidity comparable to the homogeneous benchmark Sc(OTf)_3_. Integration of ZrOTf-BTC supported on silica into a fixed-bed flow reactor enabled continuous epoxide ring-opening amination, achieving high yields of the target product (83–93%) in 30 runs and affording a total turnover number of >2700 in 30 h (Table 1, entry 3). Kute et al. [28] reported a facile ion adsorption and microwave heating method to synthesize an N-doped graphene-supported Fe single-atom catalyst (Fe_SA_@N-G) for the N-alkylation of amines with alcohols. The continuous-flow process delivered high conversion and yield for N-benzylaniline (Table 1, entry 6) under optimized conditions, and the broad applicability of this flow system was further confirmed through substrate-scope studies. Latos et al. [29] developed a highly active and stable heterogeneous catalyst by immobilizing a Lewis acidic trifloaluminate ionic liquid onto MgO-SiO_2_ supports, which exhibited good performance in the aminolysis of epoxides to afford β-amino alcohols. Integration of this catalyst into a fixed-bed reactor enabled stable operation over 72 h for the synthesis of 2-phenylamino-2-phenylethanol (Table 1, entry 7). Leung et al. [30] employed a commercially available Ni-Al_2_O_3_/SiO_2_ catalyst, which was extruded into 1 mm pellets with bentonite as a binder, for the hydrogen-borrowing amination of alcohols with ammonia. The implementation of this catalyst in a fixed-bed reactor enabled the selective synthesis of primary amines with high single-pass conversion and exclusive selectivity for most substrates (e.g., phenylmethanamine (Table 1, entry 8)), as the continuous removal of water and products effectively suppressed side reactions and catalyst deactivation. Streng et al. [34] developed a heterogeneous γ-Al_2_O_3_ catalyst for the amination of alcohols under supercritical CO_2_ (scCO_2_) conditions for the synthesis of N-alkylated heterocycles. The integration of this catalyst with a continuous-flow system featuring an automated self-optimizing reactor (utilizing SNOBFIT algorithm) allowed for real-time optimization of key parameters such as temperature and flow rate, achieving a yield up to 94% for N-methylpiperidine at 340 °C and 100 bar (Table 1, entry 12).

Noble metal catalysts, such as gold and palladium, exhibit high efficiency and selectivity in continuous-flow amination reactions under mild conditions. Nnamdi et al. [31] developed a highly stable heterogeneous Pd catalyst by immobilizing a sterically bulky Pd-PEPPSI-IPentCl complex onto silica supports, which effectively eliminates bimolecular decomposition and thereby significantly extends catalyst lifetime. The implementation of this supported catalyst in a packed-bed reactor enabled efficient Buchwald–Hartwig amination, achieving full conversion within short residence times while maintaining high selectivity and long-term operational stability (e.g., 86% yield for N-(4-methoxyphenyl)-3-(trifluoromethyl)aniline (Table 1, entry 9)). Carrillo et al. [32] developed a heterogeneous gold nanocatalyst (Au@APTES@SBA) by photochemically immobilizing Au nanoparticles onto aminopropyl-functionalized SBA-15 mesoporous silica, which exhibited high activity in the reductive amination of aldehydes with dimethylphenylsilane under mild conditions. The implementation of this catalyst in a continuous-flow packed-bed reactor enabled stable operation for approximately 18 h, achieving an accumulated turnover number (TON) of 434 and producing multigram amounts of amines with yields of 77~99% for six different amines (e.g., 85% yield and 70 TON for N-benzylaniline (Table 1, entry 10)).

Organocatalysts, such as thioureas and peptides, enable enantioselective amination in flow with good recyclability, providing a metal-free approach for sustainable synthesis. Sánchez-Molpeceres et al. [26] developed a highly efficient quinine-derived bifunctional thiourea organocatalyst featuring a 3,3-diaryl-oxindole scaffold (C1) and its linear-polymer-supported analogue (LP-IV) for the enantioselective α-amination of 4-substituted pyrazolones with azodicarboxylates. The supported catalyst LP-IV was effectively integrated into a packed-bed reactor, achieving gram-scale synthesis of chiral 4-amino-pyrazolones (Table 1, entry 4) with high yield (86%) and enantioselectivity (88:12 er), while maintaining stable performance during 3 h of continuous operation without significant loss of activity. Ötvös et al. [27] developed a heterogeneous dipeptide catalyst (H-Pro-Asp-NH-TentaGel) by immobilizing the peptide on TentaGel, a polystyrene-polyethylene glycol graft copolymer, which efficiently catalyzed the direct enantioselective α-amination of aldehydes with dibenzyl azodicarboxylate (DBAD). The integration of this supported catalyst into a packed-bed reactor achieved high conversion (83–91%) and enantioselectivity (88–92%) while maintaining stable performance over 20 h of continuous operation for the synthesis of α-hydrazino aldehydes, which were subsequently reduced to α-hydrazino alcohols (Table 1, entry 5). Kasaplar et al. [35] developed a polystyrene-supported bifunctional thiourea organocatalyst (PS-TU) through a simplified immobilization strategy for the α-amination of 1,3-dicarbonyl compounds with azodicarboxylates. The integration of PS-TU into a packed-bed reactor for the amination of ethyl 2-oxocyclopentanecarboxylate enabled stable operation over 7.5 h by implementing periodic reactivation with triethylamine washes (Table 1, entry 13), achieving a productivity of 4.88 mmol·mmol_cat_^−1^·h^−1^ and a TON of 37 while maintaining high enantioselectivity (93% ee). Alwakwak et al. [36] developed a novel metal-free trifunctional organocatalyst (Br/APS/PAIHF), which is permanently immobilized on porous polyamide-imide hollow fibers and incorporates cooperative -OH, -NH, and -Br groups for efficient amine hydroxyalkylation reactions. The integration of this catalyst into a continuous-flow system facilitated the solvent-free synthesis of aminoalcohols, such as 1-(phenylamino)propan-2-ol, with high selectivity (up to 97.5%) and improved conversion (61%) at ~5 min (Table 1, entry 14).

In solid–liquid continuous-flow amination processes, the solid phase can function not only as a catalyst but also as a reducing agent, such as picoline borane (pic-BH_3_). Gianolio et al. [33] developed a chemoenzymatic strategy where the initial decarboxylation step is catalyzed by an immobilized tyrosine decarboxylase, but the key amine functionalization is achieved through a reductive amination using picoline borane (pic-BH_3_) as a highly effective and less hazardous alternative to conventional reducing agents. The integration of the reductive amination step in a packed-bed reactor facilitated a telescoped process (Table 1, entry 11), enabling synthesis of hordenine with high conversion (92%) and 77% isolated yield during 4 h of continuous operation, while achieving a high space-time yield of 11.4 g·L^−1^·h^−1^.

### 2.3. Gas–Liquid–Solid Processes

Gas–liquid–solid amination processes, particularly reductive amination processes involving hydrogen gas as a reductant, represent a critical class of transformations driven by multiphase mechanisms, making them highly amenable to continuous-flow processing. Gaseous hydrogen and liquid substrates interact on the surface of a solid catalyst, which commonly includes noble metals (e.g., Pd, Pt, and Ru) or earth-abundant metals (e.g., Ni, Fe and Co) immobilized on supports. However, the use of hydrogen entails significant safety concerns, and reactions under ambient conditions often suffer from slow kinetics. Flow chemistry has been widely adopted to address these challenges by enhancing mass transfer, improving safety through confined hydrogen handling, and enabling precise control over reaction parameters.

*Earth-abundant metal catalysts*

The use of earth-abundant metal catalysts represents a cost-effective strategy in continuous-flow amination. As summarized in Table 2, representative examples and specific process parameters for the successful use of cobalt-, nickel-, iron-, copper-, and silver-based catalysts in gas–liquid–solid flow amination processes are provided [37,38,39,40,41,42,43,44,45,46].

Certain cobalt-based catalysts have emerged as promising earth-abundant metal alternatives to noble metals for reductive amination under continuous-flow conditions. Mi et al. [37] developed a heterogeneous cobalt catalyst supported on nitrogen-doped carbon derived from chitosan (Co@CS), enabling the efficient reductive amination of carbonyl compounds to primary amines. The integration of Co@CS into a micropacked-bed reactor achieved nearly complete conversion (>99%) and exceptional selectivity (>99%) for the synthesis of benzylamine (Table 2, entry 1), effectively suppressing the formation of secondary/tertiary amines and by-products and demonstrating stable catalytic performance through continuous operation of 40 h.

Nickel-based catalysts are widely employed in continuous-flow reductive amination processes, often demonstrating good selectivity and stability under optimized conditions. Zhang et al. [38] developed a Cs-modified Ni-Al hydrotalcite-derived catalyst (Ni_1_Al_2_-Cs_1_._0_) that achieves high activity and selectivity in the reductive amination of carbonyl compounds to primary amines under mild conditions. Integrated into a continuous-flow micropacked-bed reactor, this catalyst achieves up to 98% benzylamine yield in just 3.5 min and enables stable operation for over 500 h (Table 2, entry 2). It demonstrates promising yields under mild conditions across a broad substrate scope of carbonyl compounds. The same group [40] also developed a nickel catalyst supported on silica (Ni/SiO_2_) for the reductive amination of different carbonyl compounds. The integration of this catalyst into a micropacked-bed flow reactor exhibited superior activity and selectivity in the reductive amination of benzaldehyde (Table 2, entry 4), achieving 99% yield of benzylamine at 70 °C and 1 MPa and enabling continuous operation for 200 h with stable performance. Wang et al. [41] developed a series of silica- and alumina-supported nickel phosphide (Ni_2_P) catalysts (notably the optimized Ni_2_P/SiO_2__A600 catalyst prepared from (NH_4_)_2_HPO_4_ and reduced at 600 °C), which exhibited bifunctional properties (metal and acid sites) essential for the reductive amination of ethyl levulinate with *n*-hexylamine. The integration of this catalyst into a continuous-flow fixed-bed reactor system enabled stable operation at 10 bar and 170 °C, achieving a high N-hexyl-5-methyl-2-pyrrolidone yield of 98% with good catalyst stability over 6 h (Table 2, entry 5). Yang et al. [42] developed a highly efficient nickel catalyst supported on hydroxylapatite nanorods (10Ni-HAP), which exhibited good performance in the reductive amination of biomass-derived 2-hydroxytetrahydropyran (2-HTHP) to 5-amino-1-pentanol (5-AP) due to its high Ni dispersion, reducibility, and acidic intensity. The catalyst was implemented in a continuous-flow fixed-bed reactor, maintaining stable operation for 80 h with complete 2-HTHP conversion and a sustained 5-AP selectivity of 67–74% (Table 2, entry 6). Li et al. [43] developed a series of oxide-supported Ni catalysts, among which the Ni/ZrO_2_ catalyst exhibited superior performance in the reductive amination of 2-HTHP to 5-AP. The Ni/ZrO_2_ catalyst was tested in a continuous-flow fixed-bed reactor, allowing continuous operation for 90 h with an initial 5-AP selectivity of ~83%, which gradually decreased to ~67% (Table 2, entry 7).

Iron phosphide catalysts serve as a sustainable option in reductive amination processes. Tsuda et al. [39] developed an air-stable and highly active iron phosphide nanocatalyst supported on zirconia (Fe_2_P NC/ZrO_2_), which leverages metal-support synergy to achieve exceptional performance in the reductive amination of carbonyl compounds with H_2_ and NH_3_, exhibiting 313 times higher activity than conventional iron nanoparticle catalysts. The integration of an Fe_2_P NC/ZrO_2_ catalyst into a continuous-flow fixed-bed reactor enabled stable long-term operation over 30 h, maintaining a high yield (>80%) of benzylamine (Table 2, entry 3) under 4 MPa H_2_ at 200 °C.

Copper catalysts are efficient and selective for the synthesis of secondary amines via reductive amination. Nuzhdin et al. [44] developed a Cu-Al mixed oxide catalyst (CuAlO_x_) derived from a layered double hydroxide precursor. The integration of this catalyst into a continuous-flow fixed-bed reactor enabled efficient one-pot, two-step synthesis of secondary amines without intermediate isolation, achieving yields up to 98% for diverse substrates. Notably, in the synthesis of N-benzylaniline, the system exhibited stable operation over 6 h with a >95% yield and a space-time yield of 0.44 kg·h^−1^·L^−1^ (Table 2, entry 8), surpassing reported Pt/C systems. The same group [45] also developed an inexpensive and easily prepared copper catalyst supported on γ-alumina (Cu/γ-Al_2_O_3_). The implementation of this catalyst in a continuous-flow fixed-bed reactor enabled the synthesis of various secondary amines in high yields (up to 97%) at 110~125 °C and 50 bar H_2_. A slight deactivation of the catalyst over 2 h was observed during the reductive amination of *n*-heptanal with *p*-nitrotoluene (Table 2, entry 9).

Silver-based catalysts are also employed in reductive amination under continuous-flow mode. Artiukha et al. [46] developed a silver catalyst supported on γ-alumina (Ag/γ-Al_2_O_3_) featuring highly dispersed silver nanoparticles (mean size ~5 nm) for the one-pot reductive amination of aldehydes with nitroarenes using molecular hydrogen. The implementation of this catalyst in a continuous-flow fixed-bed reactor enabled the synthesis of diverse secondary amines in good to excellent yields (up to 92%) at 100–110 °C and 3.0 MPa H_2_. Stability testing over 2.5 h revealed that the Ag/γ-Al_2_O_3_ catalyst showed a declining yield of the secondary amine in the reductive amination of *n*-heptanal with nitrobenzene (Table 2, entry 10), and its deactivation due to carbon deposits could be completely reversed through oxidative regeneration.

*Noble metal catalysis*

Noble metal catalysts are known for their high activity and exceptional selectivity, which contributes significantly to continuous-flow amination processes. As detailed in Table 3, representative examples and specific conditions for the effective use of noble metal catalysts in gas–liquid–solid flow amination processes are summarized [47,48,49,50,51,52,53,54,55,56,57,58,59,60,61,62,63,64]. Despite concerns regarding cost and availability, noble metal catalysts remain attractive for the synthesis of high-value amine compounds because of their superior performance under mild conditions and compatibility with complex substrates.

Pd-based catalysts demonstrate strong activity and high selectivity in amination reactions, ranking among the most widely utilized systems. Zhang et al. [47] developed a highly durable palladium catalyst supported on etched silicon powder (ESi-Pd), in which Pd^0^ species are generated in situ via reduction of the Pd^II^ precursor by terminal hydrogens preloaded on the silicon surface, eliminating the need for external reductants or calcination. The integration of ESi-Pd into a continuous-flow packed-bed reactor enabled long-term reductive alkylation of amines with carbonyl compounds, achieving high yields (e.g., 85% yield for ethyl 4-(butylamino)benzoate (Table 3, entry 1)) and stable operation for over 300 h, while allowing flexible synthesis of diverse amine products within the same reactor by simply changing substrate solutions. Liu et al. [49] screened palladium hydroxide on carbon (Pd(OH)_2_/C) as an efficient catalyst for the key step of reductive amination process in the synthesis of Triflumezopyrim. The implementation of this heterogeneous catalyst in a continuous-flow fixed-bed reactor for the hydrogen reduction step of reductive amination process enabled stable operation at 70 °C and 3.0 MPa, completing the reaction within 6.5 min with 85~90% yield (Table 3, entry 3). Polidoro et al. [52] developed Pd nanoparticles supported on chitin-derived N-doped carbon materials (Pd-N/C) through two distinct synthetic strategies (Pd-N/Ca and Pd-N/Cb), yielding catalysts with different nanoparticle sizes and a homogeneous dispersion for reductive amination reactions. The implementation of the catalyst Pd-N/Ca in a continuous-flow reactor enabled efficient reductive amination of furfural with furfuryl amine, achieving full conversion and high selectivity (up to 89% toward the secondary amine product) under 30 bar H_2_ and at 25 °C (Table 3, entry 6). Saito et al. [53] developed a dimethylpolysilane-modified palladium catalyst supported on activated carbon/calcium phosphate (DMPSi-Pd/AC-CP), which exhibited high efficiency for the challenging reductive amination of nitriles with primary amines. The implementation of this catalyst in a continuous-flow fixed-bed reactor for the reductive amination of nitriles achieved moderate to high yields for several different nitriles (e.g., 91–96% yield for 2-(2-ethoxyphenoxy)-N-phenethylethan-1-amine (Table 3, entry 7)). In addition, the designed continuous-flow process enables the key C-N bond-forming step in the synthesis of (*R*)-tamsulosin, which further facilitates a four-step telescoped synthesis without intermediate isolation. Suveges et al. [57] employed a commercially available 10% palladium on carbon (Pd/C) catalyst to achieve a tandem ring-hydrogenation/reductive amination process for the synthesis of mepivacaine and its analogues. The implementation of this catalyst in a continuous-flow high-pressure system enabled the selective hydrogenation of the electron-poor pyridine ring and reductive amination of diverse substrates, delivering target amide compounds in high yields (e.g., 89% yield for bupivacaine (Table 3, entry 11)). Bana et al. [60] employed a commercially available 10% Pd/C catalyst to enable two key reductive amination steps for the synthesis of flibanserin. The integration of this heterogeneous catalyst within the continuous-flow reactors allowed the four-step sequence to operate uninterruptedly for over 2.5 h, achieving full conversion in the amination step at 100 °C and 10 bar H_2_ (e.g., reductive amination between a Boc-protected diamine and a dimethoxyacetaldehyde (Table 3, entry 14)) and delivering the target API in 31% overall isolated yield with a total residence time of less than 20 min. Ichitsuka et al. [64] developed a heterogeneous palladium catalyst system (Pd/C for phenol hydrogenation and Pd(OH)_2_/C for condensation–dehydrogenation) enhanced by styrene as a hydrogen scavenger to suppress the hydrogenation of imine intermediates during the dehydrative amination of phenols. The integration of these catalysts into a tandem packed-bed flow reactor enabled stable continuous one-week operation for the synthesis of 4-methoxy-N-phenylaniline, achieving a 96% yield of the target product with a high space-time yield of 0.31 kg·L^−1^·day^−1^ (Table 3, entry 18).

Platinum catalysts are renowned for their high activity and stability in reductive amination under demanding reaction condition. Zheng et al. [48] employed a 5% platinum on carbon catalyst (Pt/C) to catalyze a nitro reduction and reductive amination reaction. The implementation of this catalyst in a micropacked-bed flow reactor enabled a safe and efficient continuous process at 40 °C and 0.7 MPa, achieving an 87% yield and 0.26 g/h productivity for the target amine (Table 3, entry 2). Zhang et al. designed a Pt/SiO_2_ (Pt loading 1 wt%) catalyst for the selective synthesis of secondary amines from carbonyl compounds using ammonia as the nitrogen source [51]. The catalyst in the continuous-flow micropacked-bed reactor exhibited a broad substrate scope, smoothly converting diverse carbonyl compounds (including aromatic aldehydes, biomass-derived furfural, and ketone) into secondary amines (e.g., 95.5% yield for dibenzylamine (Table 3, entry 5)). Kuremoto et al. [54] employed a readily available platinum on carbon (Pt/C) catalyst packed in a continuous-flow column to achieve reductive amination between cyclohexanediamine and aromatic aldehydes (e.g., reaction between (1*R*,2*R*)-cyclohexane-1,2-diamine and benzaldehyde (Table 3, entry 8)), enabling efficient synthesis of a chiral diamine ligand library. The integration of this heterogeneous catalytic system with automated substrate feeding and fraction collection allowed rapid and reproducible production of 31 chiral diamine ligands using a single catalyst column repeatedly. Fülöp et al. [55] employed a commercially available 5% platinum on carbon (Pt/C) catalyst for the continuous-flow reductive amination of an aldehyde intermediate with 2,3-dichlorophenylpiperazine, achieving complete conversion at 80 °C and atmospheric H_2_ pressure (Table 3, entry 9). The integration of this catalyst within a continuous-flow reactor enabled its effective incorporation into a two-step consecutive system, where it followed a DIBAL-H-mediated ester reduction step. An at-line extraction was implemented between the steps to remove aluminum salts and prevent catalyst bed clogging, ultimately affording the cariprazine intermediate in 51% isolated yield. Ötvös et al. [61] employed a 5% platinum on carbon (Pt/C) catalyst supported on activated charcoal for the reductive amination step in the synthesis of the chiral key intermediate of (-)-paroxetine. The implementation of this heterogeneous catalyst in a continuous-flow fixed-bed reactor enabled a tandem reductive amination-lactamization sequence, operating stably with H_2_ gas to produce the target lactam in high yield (e.g., Table 3, entry 15) and demonstrating scalability for multigram-scale synthesis. Laroche et al. [62] employed a commercially available Pt/C catalyst for the direct reductive amination of carbonyl compounds with molecular hydrogen. The integration of Pt/C in a continuous-flow fixed-bed reactor was applied to different substrates, demonstrating good functional group tolerance and enabling rapid C-N bond formation. The continuous-flow method achieved quantitative yields and a high space-time yield of 3.9 kg/(L·day) for the synthesis of the Donepezil intermediate, while exhibiting stable long-term operation over at least 7 days with a TOF of 24 h^−1^ (Table 3, entry 16). Kobayashi et al. [63] developed and implemented a sulfur-modified platinum on carbon catalyst (3% Pt/C-S) for the continuous-flow reductive amination step in the synthesis of Safinamide mesylate. The integration of this heterogeneous catalyst into a packed-bed flow reactor enabled stable continuous operation for 72 h under optimized conditions (110 °C, 0.4 MPa), achieving a high and consistent yield of 96% and >99% ee for the target amine compound (Table 3, entry 17).

Ruthenium catalysts are a cost-effective noble metal option for reductive amination, often with good functional group tolerance and unique selectivity. Bressi et al. [50] developed a sustainable ruthenium catalyst supported on hydrochar derived from brewing industry waste (Ru-HC) for the reductive amination of biomass-derived platform molecules like levulinic acid. The integration of Ru-HC into a continuous-flow H-Cube reactor enabled efficient reaction between levulinic acid and *n*-butylamine at 80 °C and 40 bar H_2_, achieving 99% conversion and 98% selectivity (Table 3, entry 4). The substrate scoping study demonstrated that this method could convert a broad range of carbonyl compounds and amines via reductive amination, achieving 40~99% conversion of N-containing products.

Although less prevalent than other noble metals, iridium catalysts offer distinct advantages in specific applications like asymmetric reductive amination. Yasukawa et al. [56] developed PS-immobilized diamine-iridium complexes (PS-Ir D) combined with a chiral phosphoric acid (TRIP) for the asymmetric hydrogenation of imines and direct asymmetric reductive amination of ketones under hydrogen atmosphere. The implementation of this catalyst system in a continuous-flow fixed-bed reactor for the direct asymmetric reductive amination allowed stable operation for over 30 h at 0.2 MPaG, affording the tamsulosin precursor in high yield (81–95%) and enantioselectivity (91–92% ee) (Table 3, entry 10).

Gold catalysts enable the selective synthesis of secondary amines containing C=C bonds, addressing the challenge of preserving reactive double bonds during metal-catalyzed reductive amination. Nuzhdin et al. developed a 2.5% Au/Al_2_O_3_ catalyst with a mean gold particle size of 3.4 nm, prepared by deposition–precipitation for the reductive amination of aldehydes with nitroarenes using molecular hydrogen [58,59]. The implementation of this catalyst in a continuous-flow fixed-bed reactor enabled the efficient one-pot synthesis of both secondary amines [58] (e.g., reductive amination of *n*-heptaldehyde with nitrobenzene (Table 3, entry 12)) and unsaturated secondary amines [59] (e.g., reductive amination of *p*-nitrotoluene and undecylenic aldehyde (Table 3, entry 13)) with high yields at 50 bar H_2_ and 70–100 °C. Although catalyst deactivation occurred during the time-course of the reductive amination, catalytic activity was effectively restored by regeneration techniques (e.g., oxidative treatment).

## 3. Thermally Activated Amination Processes

Thermal activation can serve as a powerful and versatile strategy for driving amination reactions, serving as either an alternative or a complement to traditional catalytic pathways. By leveraging high temperature, superheated conditions, or their combination under continuous-flow mode, it is possible to achieve significant rate accelerations that reduce or even circumvent the need for catalysts. This section will explore the application of thermal activation (spanning both high-temperature (>100 °C) and superheated systems) in enabling diverse and efficient continuous-flow amination processes.

### 3.1. Non-Catalytic, Thermally Activated Amination Processes

Thermally activated amination processes discussed herein intentionally forego catalytic species, relying on the controlled harsh conditions enabled by continuous-flow systems. This approach is based on the principle that elevated temperatures can provide the necessary energy to overcome kinetic barriers, thereby driving C-N bond formation directly. This is particularly effective for reactions that are thermodynamically feasible but proceed sluggishly at conventional temperatures. By foregoing a catalyst, this approach avoids issues commonly associated with metal catalysis, such as residual metal contamination or catalyst poisoning. Continuous-flow technology is essential here, as it enables the safe, precise, and scalable implementation of harsh conditions (e.g., high-temperature/high-pressure) that are difficult to control in batch reactors. Table 4 presents typical examples where such thermally activated and non-catalytic flow processes have been implemented for amination reactions [65,66,67,68,69,70,71,72,73,74,75,76,77,78,79]. Thermally activated amination processes without catalysts primarily encompass epoxide amination, halide amination, and ester amination, among other related processes.

Aryl or alkyl halides and similar substrates undergo nucleophilic substitution with ammonia or amines to yield primary or secondary amines. Elevated temperatures and high-pressure conditions can effectively facilitate the amination reaction, and performing it under continuous-flow mode mitigates side reactions and enhances safety. Numerous studies have demonstrated that nucleophilic amination can be efficiently facilitated in readily assembled tubular reactors under high-temperature conditions by employing a variety of suitable substrates, such as alkyl/aryl halides, sulfonates, esters, and electron-deficient arenes, yielding a diverse array of N-containing products (e.g., Table 4, entries 1–4,6,7,9,10,12) [65,66,67,68,70,71,73,74,76]. Xue et al. developed a continuous-flow system utilizing a Teflon AF-2400 tube-in-tube microreactor, which safely facilitates the diffusion of ammonia from aqueous ammonia into organic reaction mixtures under elevated pressure and temperature conditions [78]. This innovative system was effectively applied to the nucleophilic amination of diverse aryl and heteroaryl halides (e.g., amination of 2-chloro-8-nitroquinoline (Table 4, entry 14)), enabling the synthesis of primary amines with high atom economy, high yields, and enhanced operational safety by avoiding the hazards associated with anhydrous ammonia gas. Flow chemistry is also commonly applied in the amination of epoxides, where these substrates undergo nucleophilic ring-opening with amines to afford N-containing products. Utilizing readily assembled tubular reactors, a range of N-containing compounds have been efficiently prepared from epoxide starting materials (Table 4, entries 8, 11, and 13) [72,75,77]. Ma et al. [69] developed a catalyst-free reductive amination system for the conversion of levulinic acid to N-substituted pyrrolidinones using formic acid as a hydrogen source in a continuous-flow microreactor (Table 4, entry 5). Tissot et al. [79] developed a stereospecific amination route for a cis-cyclobutylamine via a continuous-flow process involving the high-temperature azidation of a mesylated cyclobutanol followed directly by a Staudinger reduction (Table 4, entry 15). This continuous-flow system demonstrated a key advantage for amination reactions by safely enabling high-temperature conditions to achieve effective conversion while avoiding the isolation of a potentially explosive alkyl azide intermediate, thereby significantly enhancing process safety.

### 3.2. Catalytic, Thermally Activated Amination Processes

Although thermal activation provides a degree of rate acceleration, catalysts are usually required to drive reactions to high conversion with good selectivity far more efficiently. In catalytic amination under high-temperature flow conditions, the combination of thermal energy and a catalyst creates a synergistic effect. Both contribute to overcoming inherent kinetic barriers, and the catalyst additionally directs the reaction pathway to ensure precise molecular construction. Although Section 2 has already presented many cases of catalytic, thermally activated amination processes in heterogeneous systems, additional cases (liquid-phase systems) are provided here to further elaborate on the topic (Table 5) [80,81,82,83,84,85].

Chauhan et al. [80] developed a CuBr_2_/NMO catalytic system for the α-amination of esters. Under continuous-flow mode, this method delivers α-amino esters in moderate to good yields (e.g., 83% yield for ethyl 2-phenyl-2-(piperidin-1-yl)acetate (Table 5, entry 1)) at 100 °C and 25 min, with the catalytic cycle maintained by NMO-mediated oxidation of Cu(I)Br to regenerate active Cu(II)Br in situ. Heider et al. [81] developed a homogeneous Ru-based catalytic system, utilizing [Ru(*p*-cymene)Cl_2_]_2_ in combination with Xantphos as a ligand, facilitating the amination of alcohols through the borrowing hydrogen mechanism. The integration of this catalyst with continuous-flow technology in a tubular reactor enabled operation at 290 °C and 15 min under high pressure, leading to 97% conversion of 1-octanol and high catalytic activities with turnover frequencies exceeding 750 h^−1^ (Table 5, entry 2). Lai et al. [82] developed a highly efficient iron(II) chloride hydrate (FeCl_2_·*n*H_2_O) catalytic system for the amination of sulfides and sulfoxides using N-mesyloxycarbamates for the synthesis of sulfilimines and sulfoximines. By employing 1-butylimidazole as a base to ensure homogeneous reaction mixtures, the application of this catalyst in continuous-flow processes significantly reduced reaction times to 1–10 min at 80–100 °C and provided high yields for a broad substrate scope (e.g., 95% sulfilimine yield in Table 5, entry 3). Wong et al. [83] employed a recyclable palladium precatalyst, specifically *t*-BuXPhosPd(π-cinnamyl)OTf, which facilitates C-N bond formation between amines and (hetero)aryl bromides with low catalyst loadings. The integration of this catalyst into a continuous-flow system utilizing water as the primary reaction medium (e.g., amination between 4-bromoacetophenone and 4-methoxyanilne in Table 5, entry 4) achieved rapid aminations with excellent efficiency, reduced waste (low *E* factors), and recyclability of both the aqueous medium and catalyst. Bédard et al. [84] constructed a versatile, reconfigurable continuous-flow system integrated with BrettPhosPdG_3_ precatalyst for Buchwald–Hartwig amination, enabling automated optimization through real-time analytics and modular reactor design. The system demonstrated high efficiency in C-N bond formation, achieving high yield for diverse amine substrates (e.g., 72% yield for bis(4-methoxyphenyl)amine (Table 5, entry 5)) under autonomously optimized conditions, while allowing rapid scalability and remote monitoring via a user-friendly interface. Chen et al. [85] developed a superacidic reagent system utilizing trimethylsilyl azide (TMSN_3_) and triflic acid (TfOH) for the direct introduction of amino groups via electrophilic amination of arenes and Schmidt reactions of carboxylic acids. The implementation of this system in a continuous-flow setup allowed for safe handling of hazardous intermediates, achieving rapid reaction completion within 2–5 min at 90 °C and providing good to excellent yields (e.g., 78% yield for toluidines (Table 5, entry 6)) for a diverse range of substrates.

**Figure 2:**
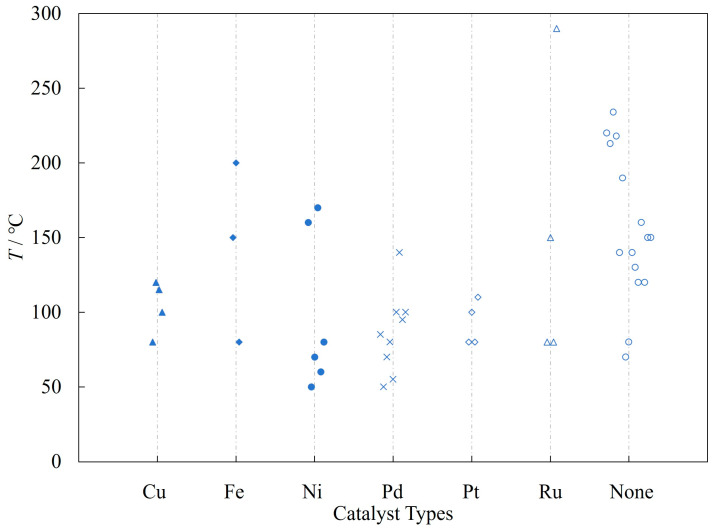
Reaction temperature distribution for catalyzed and non-catalyzed amination processes.

Figure 2 summarizes the distribution of reaction temperatures for typical catalyzed and non-catalyzed amination cases. This analysis considers typical metal catalysts (noble metals: Pd, Pt, and Ru; earth-abundant metals: Cu, Fe, and Ni) and catalyst-free conditions, based on reaction cases at ≥50 °C from Section 2 and Section 3. Optimal amination performance is contingent upon the judicious selection of a reaction temperature that is compatible with the specific catalyst system employed. Metal-catalyzed amination processes typically occur at temperatures below 200 °C, whereas non-catalyzed amination reactions often require significantly higher temperatures to drive the reaction forward.

## 4. Enzymatic Amination Processes

Enzymatic amination in continuous-flow systems represents an important research direction toward sustainable and efficient chemical synthesis, offering distinct advantages over conventional metal-catalyzed approaches. By leveraging the inherent selectivity of enzymes, this methodology avoids the use of toxic heavy metal catalysts and harsh reaction conditions, aligning with green chemistry principles. The continuous-flow method achieves effective process intensification by enabling precise control over reaction parameters, minimizing product inhibition, and maintaining consistent catalyst performance over time through immobilization. Unlike metal-catalyzed amination processes, which often require harsh conditions and carry the potential environmental burden, enzymatic amination processes operate under mild aqueous conditions, typically with water as the primary by-product. This approach can simplify downstream purification and provides inherent stereoselectivity that reduces the need for chiral ligands or costly separation steps. The integration of continuous-flow biocatalysis thus offers a promising platform toward robust, scalable, and environmentally benign synthesis of amines. Researchers have investigated and reported numerous continuous-flow enzymatic amination processes [86,87,88,89,90,91,92,93,94,95,96,97,98,99,100], with typical examples summarized in Table 6.

### 4.1. Transaminase-Catalyzed Processes

Transaminases are key biocatalysts enabling the green and efficient synthesis of amines through a pyridoxal phosphate (PLP)-dependent transamination. In continuous-flow systems, immobilized transaminases in packed-bed reactors overcome typical batch limitations, enabling enhanced productivity, operational stability, and easier integration with downstream processing.

Božinović et al. [86] developed a highly efficient biocatalytic system employing the ω-transaminase *N*-His_6_-ATA-wt, which achieved a 96% yield within 30 min in the transamination of furfural to furfurylamine using (*S*)-(-)-α-methylbenzylamine as an amine donor under mild conditions. The enzyme immobilized on functionalized magnetite nanoparticles was evaluated in a continuous-flow 3D-printed magnetic field-assisted microreactor over 18 days of continuous operation, achieving a maximum space-time yield of 1.07 g·L^−1^·h^−1^ and a total turnover number of 2.04 × 10^7^ (Table 6, entry 1). Heinks et al. [87] developed an immobilized amine transaminase from *Silicibacter pomeroyi* (ATA-Spo) on glutaraldehyde-functionalized amine beads, exhibiting high activity and stability for reuse in the reductive amination of 5-(hydroxymethyl)furfural. The implementation of this immobilized biocatalyst with isopropylamine in a continuous-flow packed-bed reactor enabled continuous operation for 12 days with a gradual decrease in conversion from 75% to 41% (Table 6, entry 2). Padrosa et al. [93] developed a novel alanine dehydrogenase from *Halomonas elongata* (HeAlaDH) combined with the formate dehydrogenase from *Candida boidinii* (CbFDH), enabling efficient recycling of L-alanine as an amino donor in transaminase-catalyzed amination reactions. The implementation of the immobilized enzymes in a packed-bed reactor with HeWT transaminase and co-immobilized HeAlaDH/CbFDH achieved up to 40% conversion of vanillin with 20 min retention time using Ep-Ag support (Table 6, entry 8). Böhmer et al. [94] developed an efficient immobilized ω-transaminase biocatalyst by immobilizing (*R*)-selective ω-transaminase from *Arthrobacter* sp. (AsR-ωTA) or (*S*)-selective ω-transaminase from *Chromobacterium violaceum* (Cv-ωTA) on an EziG carrier for the amination of 1-phenoxypropan-2-one with 2-propylamine. The implementation of the immobilized biocatalyst (EziG_3_-AsR-ωTA) in a continuous-flow packed-bed reactor with toluene served as the solvent, achieving ~70% conversion with a space-time yield of 1.99 g·L^−1^·h^−1^ in 72 h and >90% conversion in 120 h (Table 6, entry 9). Eimear Hegarty and Francesca Paradisi [97] exploited an (*S*)-selective transaminase from *Halomonas elongata* (*He*WT), which efficiently catalyzes the reductive amination of cyclic ketones to their corresponding small cyclic amines. The implementation of this enzyme, covalently immobilized on an epoxy support, in a continuous-flow packed-bed reactor enabled high substrate conversion within short residence time (e.g., 98% conversion of tetrahydrofuran-3-one in 5 min (Table 6, entry 12)).

### 4.2. Oxidoreductase-Catalyzed Processes

Reductive amination catalyzed by oxidoreductases can provide a direct and atom-economical route to enantiomerically pure amines. These enzymes require the reduced nicotinamide cofactor NAD(P)H to drive the reductive step, making efficient cofactor regeneration essential for practical synthesis. The implementation of these biocatalysts in immobilized form within continuous-flow packed-bed reactors can significantly enhance amination processes by facilitating cofactor recycling, improving operational stability, and increasing overall productivity compared to traditional batch systems.

Croci et al. [88] developed a co-entrapped enzyme catalyst system comprising an amine dehydrogenase (LE-AmDH-v1) and a formate dehydrogenase (Cb-FDH) immobilized within an agarose hydrogel, catalyzing the reductive amination of aldehydes under mild aqueous conditions. The implementation of this biocatalyst in a continuous-flow reactor via 3D-printing technology enabled sustained operation for 120 h, achieving a 47% analytical yield and a space-time yield of 7.4 g·L^−1^·day^−1^ in the conversion of benzaldehyde to benzylamine (Table 6, entry 3). Franklin et al. [89] developed a co-immobilized chimeric amine dehydrogenase (AmDH) and formate dehydrogenase (FDH) catalyst on Nuvia^®^ IMAC resin, catalyzing the reductive amination of 5-methyl-2-hexanone to enantiomerically pure (*R*)-amines. The implementation of this biocatalyst in a continuous-flow packed-bed reactor enabled stable operation for >24 h. In addition, conversion of 5-methyl-2-hexanone (20 mM) can achieve a maximum conversion of 48%, with a volumetric productivity of 166 g·L^−1^·day^−1^ at the residence time of 11.9 min (Table 6, entry 4). Finnigan et al. [90] developed an immobilized two-enzyme system comprising a reductive aminase from *Ajellomyces dermatitidis* (*Ad*RedAm) and a glucose dehydrogenase from *Bacillus subtilis* (*Bs*GDH) on an EziG^3^ carrier for the biocatalytic reductive amination. The implementation of this biocatalyst in a continuous-flow fixed-bed reactor, optimized via a hybrid mechanistic and empirical modeling approach, achieved 98% conversion, 44% isolated yield, a space-time yield of 10.3 g·L^−1^·h^−1^, and 132 h of stable operation under the scenario of low waste (Table 6, entry 5). Xie et al. [92] developed a co-immobilized enzyme catalyst system consisting of amine dehydrogenase wh84 and glucose dehydrogenase on epoxy resin LXTE-706 carriers for the asymmetric reductive amination of 1-hydroxybutan-2-one to (*S*)-2-aminobutan-1-ol. The implementation of this biocatalyst in a continuous-flow packed-bed reactor achieved 99% conversion and 99% ee with a space-time yield of 124.5 g/(L·day), while maintaining an average conversion of 91.8% over 48 h of continuous operation (Table 6, entry 7). Chen et al. [95] developed the co-immobilized enzyme system stably anchored on dendritic organosilica nanoparticles (*Ja*AmDH&GDH@DON) for the enantioselective amination of ketones to chiral amines. This integration of the immobilized biocatalysts with the Ru-NHC@DON-catalyzed alcohol oxidation module into the continuous-flow packed-bed reactor established an efficient chemoenzymatic cascade. This process achieved high yield and enantioselectivity in the synthesis of chiral amines from racemic alcohols (e.g., 97% yield and 99% ee for (*R*)-1-phenylethan-1-amine (Table 6, entry 10)). Mattey et al. [96] investigated a series of enzyme catalysts, including alcohol oxidases, transaminases, and reductive aminases, which were individually immobilized to enable previously incompatible multi-enzyme cascades for amine synthesis. The integration of these biocatalysts into modular continuous-flow packed-bed reactors, combined with a multipoint injection reactor for efficient oxygen supply, allowed the successful synthesis of diverse primary and secondary amines, including the natural product 4O-methylnorbelladaine through oxidase-RedAm cascade (Table 6, entry 11). Huang et al. [98] developed an integrated chemoenzymatic cascade system employing an organo-enzymatic decarboxylative fluorination (CALB) and a bienzymatic reductive amination module (AmDH/GDH) for the enantioselective synthesis of chiral α-mono- and difluoromethyl amines from β-keto-acid esters. The implementation of this immobilized enzyme system in the continuous-flow packed-bed reactor not only achieved a high space-time yield of 17.1–19.7 g·L^−1^·h^−1^ but also maintained 85–98% yield during 96 h of continuous operation for a model substrate of ethyl 3-oxo-3-phenylpropanoate (Table 6, entry 13). In addition, the continuous-flow system demonstrated broad substrate scope, converting various β-keto-acid esters to chiral α-monofluoromethyl amines in 34–98% yield with 99% ee at 100 mM substrate concentration. Kong et al. [99] developed a modular immobilized multienzyme catalyst system for enantioselective benzylic C-H amination of arylalkanes. The system comprised two bienzymatic modules, namely an *Aae*UPO-GO module for C-H hydroxylation and a *Cps*ADH-*Ja*AmDH module for alcohol amination. The implementation of this catalyst system in the continuous-flow packed-bed reactor achieved 96% yield and a space time yield of 3.2 g·L^−1^·h^−1^ for the substrate of ethylbenzene at the 50 mmol scale (Table 6, entry 14), while demonstrating broad substrate scope across various aromatic alkanes.

### 4.3. Hydrolase-Catalyzed Processes

Within the realm of enzymatic amination under continuous-flow conditions, hydrolytic enzymes such as lipases offer a distinct and valuable approach, primarily catalyzing aminolysis reactions for the synthesis of amides. The flow-based strategy effectively overcomes common batch limitations, enabling efficient chemoselective transformations and facilitating complex operations like dynamic kinetic resolution. Andrade et al. [91] employed a confined lipase from *Candida antarctica* (CAL-B) to achieve highly efficient aminolysis reactions for the synthesis of diverse carboxamide derivatives under continuous-flow conditions. The implementation of CAL-B in a packed-bed flow reactor significantly enhanced reaction efficiency, achieving 98% conversion for the synthesis of N-butyloctanamide in 10 min at 50 °C while maintaining stable performance over 84-fold residence time without loss of enzymatic activity (Table 6, entry 6). Falus et al. [100] developed the immobilized Alcalase (Subtilisin A) biocatalysts adsorbed onto surface-grafted macroporous silica gels, which demonstrated high activity and enantioselectivity in the kinetic resolution of racemic N-Boc-phenylalanine enthyl thioester with benzylamine. The integration of these biocatalysts into the continuous-flow packed-bed reactor system, comprising alternating packed-bed enzyme columns (at 50 °C for KR) and racemization columns (at 150 °C for racemization), enabled efficient dynamic kinetic resolution, achieving 79% conversion and 98% enantiomeric excess for the (*S*)-amide product with a volumetric productivity of 8.17 g·L^−1^·h^−1^ (Table 6, entry 15).

## 5. Alternative Energy-Driven Amination Processes

The application of flow chemistry in amination reactions has garnered significant attention due to its ability to enhance reaction efficiency, safety, and sustainability. Within this field, the utilization of alternative energy sources, such as light and electricity, represents a promising frontier. Flow chemistry offers distinct advantages for alternative energy-driven amination by enabling precise control over reaction parameters, facilitating the use of traceless reagents and improving scalability. This section will focus on photo- and electricity-driven amination processes, highlighting how continuous-flow systems overcome limitations of batch processes.

### 5.1. Photo-Driven Amination Processes

Photo-driven amination processes benefit immensely from flow chemistry technology, as the high surface-to-volume ratio of microreactors ensures uniform irradiation and effectively mitigates the light attenuation issues described by the Beer–Lambert law. This leads to accelerated reaction rates, reduced formation of by-products, and reliable handling of photolabile intermediates. The integration of continuous-flow technology allows for the efficient use of photoredox catalysts and light sources, enabling sustainable amination under mild conditions.

**Figure 3:**
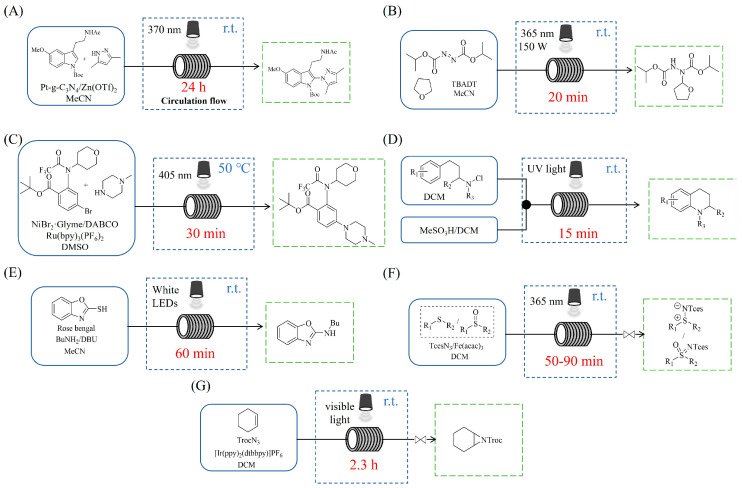
Continuous-flow processes for the photo-driven amination reactions. (**A**) Synthesis of an intermediate for the melatonin derivative. (**B**) TBADT-mediated C(sp^3^)-H amination. (**C**) C-N coupling via photo-redox. (**D**) Photochemical amination of N-chloramines. (**E**) Photocatalytic amination of 2-mercaptobenzoxazole. (**F**) Iron-catalyzed amination of sulfides/sulfoxides with TcesN_3_. (**G**) Photocatalytic aziridination of cyclohexene.

Shi et al. [101] developed a photocatalytic hydrogen-evolution cross-dehydrogenative coupling system using a Pt-g-C_3_N_4_ single-atom catalyst for the direct C(sp^2^)-N bond formation between various (hetero)arenes and nitrogen-containing nucleophiles under 370 nm light irradiation. The integration of a simple, in-house-built high-speed circulation flow system enabled the scalable decagram scale synthesis of amination products, such as 65% yield for the intermediate of a melatonin derivative (Figure 3A). Wen et al. [102] developed a tetrabutylammonium decatungstate(TBADT)-mediated photocatalytic hydrogen atom transfer process for the C(sp^3^)-H amination under 365 nm light irradiation. The integration of a continuous-flow system with in-line organic solvent nanofiltration enabled highly efficient catalyst recovery and recycling, achieving a remarkable turnover number (TON) of over 8400 for the amination reaction (Figure 3B). Cordell et al. [103] developed NiBr_2_-mediated photo-redox catalyzed C-N cross-coupling for a key amination step in the total synthesis of entrectinib, utilizing blue light (405 nm LEDs) to drive the reaction under mild conditions. The implementation of continuous-flow photochemistry facilitated the synthesis of the critical intermediate at 50 °C and 30 min with 81% conversion to the desired coupled product and 50% isolated yield (Figure 3C). Cosgrove et al. [104] developed direct photochemical C-H amination of arenes utilizing aminium radicals generated from the photolysis of N-chloroamines under UV light. The implementation of the photochemical amination in a continuous-flow reactor (Figure 3D) not only overcomes the scalability limitation of the batch process but also enables the integration of in-line N-chloroamines generation, allowing for the continuous and direct transformation of secondary amines into their arylated derivatives. Rattanangkool et al. [105] developed metal-free, organocatalytic visible-light-driven amination via a photochemical S_N_Ar pathway using Rose Bengal as the photocatalyst to convert heterocyclic thiols into valuable 2-aminobenzoxazoles and 4-aminoquinazolines under mild conditions with white LED irradiation. The adoption of a continuous-flow photoreactor, specifically a transparent PFA tubular reactor, enabled complete reaction conversion for the amination of 2-mercaptobenzoxazole within a short residence time of 60 min (Figure 3E). Lebel et al. [106] developed a continuous-flow process for iron-catalyzed photochemical amination of sulfides and sulfoxides with azides using UVA light (365 nm) and an Fe(acac)_3_ catalyst to produce sulfilimines and sulfoximines at room temperature with short residence time (Figure 3F). The implementation of the purpose-built continuous-flow photochemical reactor, specifically a capillary cylinder design, was crucial for enhancing light exposure efficiency, ensuring safer handling of potentially hazardous azide reagents, and enabling facile gram-scale synthesis with high productivity. Scholz et al. [107] developed visible-light-driven photocatalytic aziridination of alkenes with azidoformates as nitrene precursors. The reaction proceeds via triplet energy transfer from an iridium sensitizer to selectively generate triplet nitrenes, thereby avoiding competitive side reactions. The implementation of a continuous-flow reactor effectively addressed the photon-limited nature of this photochemical process (Figure 3G), enabling significant scalability, reduced azide equivalents, and the preparative-scale synthesis of aziridines with high efficiency.

### 5.2. Electricity-Driven Amination Processes

Electricity-driven amination reactions are similarly enhanced in continuous-flow systems, where the confined geometry ensures efficient electron transfer and often eliminates the need for stoichiometric oxidants or reductants. Flow electrochemistry provides superior control over current density and residence time, reducing electrode fouling and enabling the safe use of hazardous reagents. The scalability and modularity of flow reactors make electrochemical amination an attractive option for synthesizing complex amines with high selectivity.

**Figure 4:**
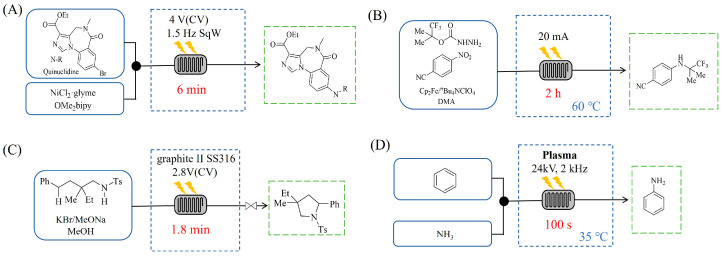
Continuous-flow processes for the electricity-driven amination reactions. (**A**) C-N functionalization of bromo-Flumazenil. (**B**) Electrochemical deoxygenative amination. (**C**) Electrochemical C(sp^3^)-H amination. (**D**) Direct amination of benzene with ammonia.

Morvan et al. [108] developed electrochemical C-N arylation for compound library synthesis using alternating polarity, which enables amination of diverse aryl halides with a broad range of amines in air and without supporting electrolytes. The implementation of this alternating polarity in a continuous-flow electrochemical reactor was essential to eliminate electrode passivation, ensure high reproducibility over consecutive runs, and facilitate automated synthesis of substantial diverse C-N arylation products (e.g., C-N functionalization of bromo-Flumazenil in Figure 4A). Xu et al. [109] established a versatile electrochemical strategy that enables deoxygenative amination using alcohol-derived carbazates and nitroarene for the synthesis of alkylamines (primary, secondary, and tertiary). The electrochemical continuous-flow system allowed the target amination product to be synthesized in just 2 h, significantly reducing the reaction time from 6 h required in batch (Figure 4B), and the flow system enabled synthesis on a 10 mmol scale with 71% yield in 20 h. Nikolaienko et al. [110] developed electrochemical intramolecular C(sp^3^)-H amination via remote hydrogen atom transfer to synthesize pyrrolidines under mild conditions without stoichiometric oxidants. The implementation of a continuous-flow electrochemical reactor enabled efficient preparation of the desired pyrrolidine by addressing the issue of hydrogen gas evolution via pressure regulation, allowing for consistent reaction performance over 34 h with 76% isolated yield (Figure 4C). Besides the direct application of electricity in electrochemical amination processes, electricity can also be harnessed to generate plasma for facilitating amination reactions through plasma chemistry. Dupont et al. [111] developed a catalyst-free direct amination of benzene with ammonia utilizing a microreactor, where the plasma activation of ammonia generates reactive amino radicals that enable single-step aniline synthesis under mild conditions. The implementation of a specialized gas–liquid flow microreactor was essential to maintain a stable NH_3_ plasma–liquid reactant interface and prevent unwanted by-products, thereby enhancing selectivity towards aminated products like aniline (Figure 4D). This flow system also demonstrated versatility by enabling the amination of other substrates such as alkenes.

## 6. Other Enabling Scenarios for Flow Amination Processes

Beyond the primary application scenarios categorized above, flow chemistry also unlocks several other distinct advantages in amination processes that merit dedicated discussion. These include enhancing reaction safety, enabling precise control over mixing-sensitive steps, and facilitating the integration of amination into complex multi-step sequences. It is important to note that these application scenarios are not strictly isolated. They often intersect and complement each other, demonstrating the versatility and integrative power of continuous-flow systems.

### 6.1. Safety-Enhanced Processes

Flow chemistry significantly enhances safety in amination reactions by confining hazardous reagents or intermediates within small reactor volumes. Continuous-flow systems allow for the on-demand generation and immediate consumption of toxic or unstable compounds, minimizing exposure risks. Continuous-flow systems allow for the safe handling of volatile materials under pressurized conditions, coupled with inline quenching and real-time monitoring to prevent accidents. This approach makes flow chemistry particularly valuable for industrial applications where amination steps involve high-energy intermediates or strongly exothermic processes.

**Figure 5:**
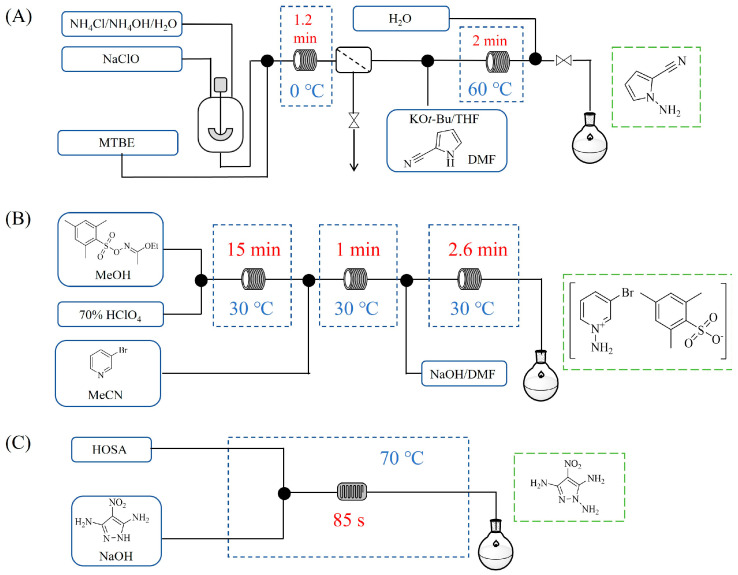
Continuous-flow processes for the enhanced safety. (**A**) Designed process involving monochloramine preparation and amination step. (**B**) In situ preparation and consumption of MSH for amination of 3-bromopyridine. (**C**) N-amination of nitrogen-rich compound 3,5-diamino-4-nitropyrazole.

Vasilev and colleagues [112] developed a continuous-flow process to synthesize a key pyrrolo[2,1-*f*][1,2,4]triazin-4-amine intermediate for Remdesivir, specifically addressing the significant safety risks associated with the batch N-amination step which employed hazardous sodium hydride (NaH) in dimethylformamide. By transitioning to a flow system, they replaced NaH with the safer, soluble base potassium tert-butoxide (KO*t*-Bu) and implemented on-demand, in situ generation of unstable monochloramine, thereby eliminating the need to handle or store the hazardous reagent (Figure 5A). This approach not only markedly enhanced process safety by containing reactive intermediates in a small volume and enabling precise process control but also achieved a high-yielding, scalable, and reproducible synthesis suitable for manufacturing the vital antiviral drug component. Brocklehurst et al. [113] demonstrated a continuous-flow strategy to safely handle the highly unstable and potentially explosive aminating reagent O-mesitylsulfonylhydroxylamine (MSH). By preparing MSH in situ from acetimidate and perchloric acid in acetonitrile and immediately consuming it in a subsequent pyridine amination step within the flow system (Figure 5B), they circumvented the need to isolate, store, or handle the neat hazardous solid. Tan et al. [114] addressed the inherent safety and efficiency challenges of the traditional batch N-amination process for nitrogen-rich energetic compounds, which employs hazardous reagents and involves prolonged reaction times. They developed a continuous-flow strategy using a heart-shaped microreactor (Figure 5C), which enabled effective mixing and precise control over parameters like temperature and residence time, thereby safely confining and managing the hazardous materials. This approach significantly enhanced process safety, drastically reduced the reaction time, and simultaneously increased the yield of the amination products.

### 6.2. Mixing-Sensitive Processes

**Figure 6:**
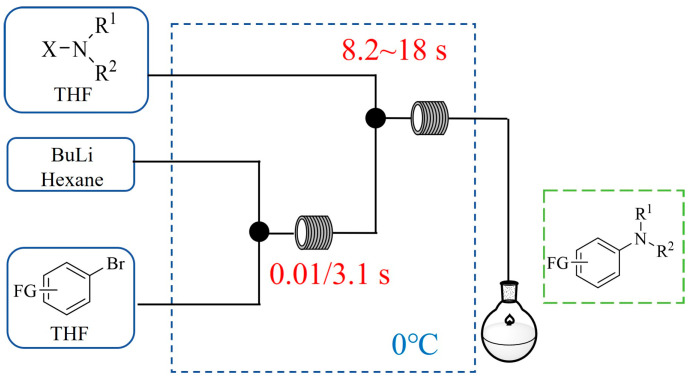
Continuous-flow process for the amination of various functionalized aryllithiums.

Mixing-sensitive amination reactions, such as those involving fast kinetics or competing pathways, are optimally performed in flow reactors due to their superior mixing efficiency. Microstructured mixers can ensure rapid and homogeneous reagent combination, reducing by-product formation and improving selectivity. In addition, the precise control over residence time in the continuous-flow reactor allows for the interception of short-lived intermediates, enabling transformations that are impractical in batch. This capability is crucial for reactions like organometallic aminations, where mixing effect dictate outcomes. Kim et al. [115] addressed the challenge of performing catalyst-free, electrophilic amination of highly reactive and unstable functionalized organolithiums, a transformation that is highly sensitive to mixing efficiency and reaction time to prevent undesired side reactions. By employing flow microreactors, they achieved precise control over the extremely short reaction time (within 20 s) at 0 °C (Figure 6), enabling efficient C-N bond formation with a sterically hindered aminating reagent. This work shows how flow technology is uniquely suited for mixing-sensitive amination steps, facilitating not only rapid optimization of reaction process but also the successful integration of reagent generation and reaction in a single streamlined process.

### 6.3. Integration of Multi-Step Flow Processes

**Figure 7:**
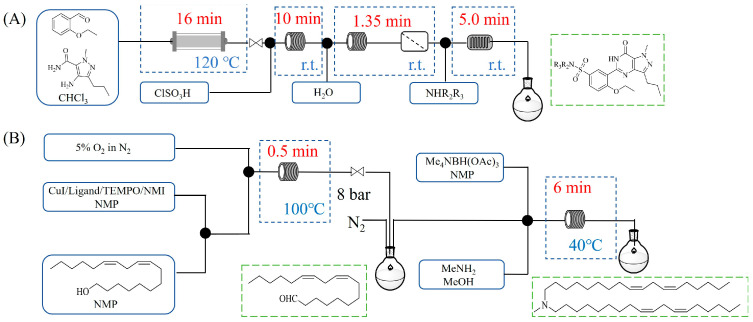
Continuous-flow processes for the multi-step integration. (**A**) Integrated process for PDE-5 API synthesis. (**B**) Integration of the oxidation and reductive amination steps.

The integration of an amination step into multi-step continuous-flow processes exemplifies the power of flow chemistry for complex synthesis. Telescoped reactions eliminate intermediate isolation, reducing solvent use and processing time. Flow systems facilitate the seamless integration of amination with upstream or downstream processes by incorporating inline separation and purification technologies. This approach can streamline the production of pharmaceuticals and fine chemicals, thereby enhancing overall efficiency and sustainability. The modularity of flow reactors allows for flexible reconfiguration, accommodating diverse synthetic routes with high reproducibility. Sthalam et al. [116] developed a fully integrated continuous-flow platform for the synthesis of PDE-5 inhibitors, which seamlessly combines cascade condensation–cyclization, in situ sulfonation, and final amination steps into a single continuous-flow process (Figure 7A). The amination step, involving the reaction of a sulfonated intermediate with *N*-methyl piperazine, was optimized to proceed efficiently at ambient temperature with a 94% yield in just 5 min residence time. The integrated multi-step flow process achieves the synthesis of sildenafil with a 65% overall yield and a productivity of 5.7 g/day within approximately 32 min, constituting a substantial enhancement in efficiency and productivity over traditional batch methods. Fujiwara et al. [117] developed an integrated two-step continuous-flow process for synthesizing the cationic lipid SST-01, a key component of siRNA-lipid nanoparticles, starting from inexpensive linoleyl alcohol. Their work combined safe, homogeneous Cu^I^/TEMPO-catalyzed aerobic oxidation using diluted oxygen with a subsequent reductive amination step, meticulously optimizing the solvents, reagents, and mixing order to maintain homogeneity and prevent clogging. This two-step flow process achieved the target lipid amine in 95% yield (76% isolated yield) with a productivity of 2.2 g/h (Figure 7B), demonstrating a more efficient and scalable route compared to the batch methods.

## 7. Process Investigation for Flow Amination Reactions

### 7.1. Novel Flow Process Development

The transition of amination processes from batch to continuous-flow mode offers significant potential for process intensification, improved safety, and enhanced sustainability. However, realizing this potential requires overcoming specific engineering and chemical challenges inherent to continuous operation. This section delves into key innovative strategies developed to address these challenges, moving beyond simple translation of batch protocols to create practical flow processes. It explores the handling of solid-containing reactions, the application of 3D printing for bespoke reactor components, innovative methods for homogeneous catalyst recycling, and the application of magnetic fields for catalyst immobilization. Furthermore, it highlights the critical role of quantitative process evaluation, employing tools like Life Cycle Assessment (LCA), to validate the environmental and economic benefits of developed novel flow processes.

*Solid Handling*

**Figure 8:**
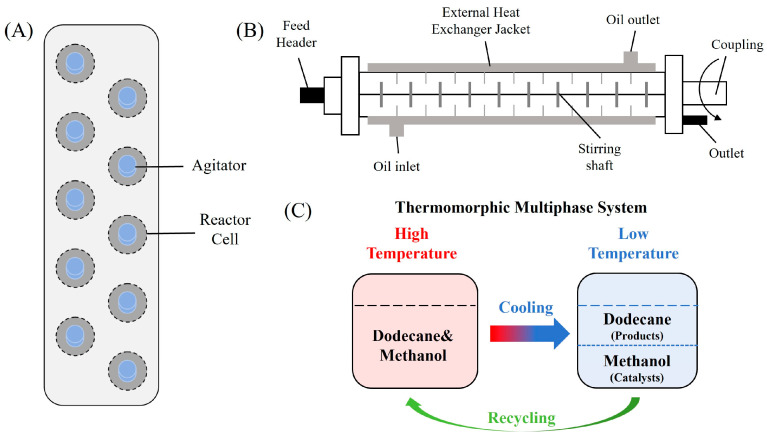
The developed novel flow processes for amination reactions. (**A**) The Coflore agitating cell reactor (ACR). (**B**) The agitated tube reactor (ATR). (**C**) Thermomorphic multiphase system.

Solid handling represents a significant challenge in the development of continuous-flow amination processes, as the formation, transport, and management of solids (including reactants, intermediates, products, or by-products) can lead to clogging, fouling, and unstable operation. Effective solutions are therefore critical for realizing robust and scalable flow amination processes. The commercial Coflore^®^ ACR (Agitated Cell Reactor) (Figure 8A) employs an external agitation platform to impart lateral motion to a reactor block containing multiple cells in series, thereby generating mechanical mixing within each cell to handle the amination processes involving solid phases [118,119]. The work by Guo et al. [118] on the synthesis of remdesivir’s nucleobase unit exemplifies a comprehensive, multi-step flow synthesis where solid handling was integral. Their process incorporated dedicated in-line units of continuous-flow solid filters to remove solids that would clog downstream reactors and employed specialized reactors of the Coflore ACR to process slurries containing insoluble reagents like NaH, enabling a fully five-step continuous-flow sequence. In a separate study, Luo et al. utilized the Coflore ACR for the amination of 4-fluoronitrobenzene with methylamine [119]. The ACR’s lateral shaking agitation effectively kept the forming product (N-Methyl-4-nitroaniline) suspended, preventing blockages despite high solid content, and even yielded a product with a favorable, small particle size distribution directly from the reactor. In the synthesis of a quinazoline intermediate for Gefitinib and Larotinib, Zhang et al. employed the agitated tube reactor (ATR, Figure 8B) to handle suspensions of poorly soluble starting materials and intermediates during chlorination and amination steps [120]. The ATR operates with an internal stirring shaft to agitate the reaction mixture, enabling efficient mixing of gases, liquids, and fine solids while minimizing back-mixing, which significantly enhances mass and heat transfer for homogeneous and heterogeneous processes. A continuous-flow process comprising three chemical transformations for the synthesis of the key API intermediate hydroxyquinazoline from acetylquinazolinone was achieved using three ATR units connected in a continuous flow setup, delivering an overall yield of 85.1% for target product and a throughput of 10.89 g/h without intermediate isolation.

*Three-dimensional printing*

Three-dimensional printing has emerged as an important tool for the design and fabrication of novel, bespoke reactor components in flow amination processes, offering exceptional geometric freedom, rapid prototyping, and the fabrication of structures optimized for enhanced mixing, heat/mass transfer, and catalyst integration. Genet et al. demonstrated the use of additively manufactured, catalytically active static mixers (CSMs) for heterogeneous reductive amination [121]. Three-dimensionally printed stainless steel mixer scaffolds were coated with a catalytically active layer of either Pd(0) (via electroplating) or Ni(0) (via cold spraying) and fitted inside a tubular flow reactor. This system enabled efficient reductive amination of various aldehydes and ketones with high conversions through a single-step flow procedure. For less reactive substrates, a two-step continuous process involving separate imine formation and catalytic reduction stages was developed to improve overall conversion, showcasing the flexibility of the modular flow setup. In a biocatalytic approach, Croci et al. utilized 3D printing to create custom molds for enzyme immobilization [88]. They co-entrapped an amine dehydrogenase (LE-AmDH-v1) and a formate dehydrogenase (Cb-FDH) within an agarose hydrogel, which was cast inside the 3D-printed mold to form a structured reactor insert with internal flow channels. This bioreactor was used for the continuous-flow reductive amination of benzaldehyde, operating for 120 h and achieving a significant product yield. This method proved superior to alternative enzyme immobilization techniques (e.g., covalent or affinity binding) for their system, as it prevented substrate adsorption and enzyme leaching under operational conditions.

*Catalyst Separation*

Phase-transition-based catalyst separation provides an effective solution for recycling expensive homogeneous catalysts in continuous-flow amination processes, utilizing solvent systems that switch between a single homogeneous phase at reaction temperature and a biphasic system upon cooling, thereby enabling efficient product–catalyst partitioning. As illustrated in Figure 8C, the amination reaction employs a thermomorphic multiphase system (TMS) of dodecane and methanol as solvents, as reported in the literature [122,123]. Bianga et al. [122] demonstrates the transfer of catalytic autotandem reactions into continuous-flow processes, specifically for the homogeneously catalyzed hydroaminomethylation (a tandem hydroformylation/reductive amination sequence) to synthesize amines from alkenes. They rationally developed a TMS using methanol and *n*-dodecane, which provided a homogeneous reaction medium at 125 °C for the Rh/SulfoXantphos catalyst and spontaneously separated upon cooling, allowing catalyst recycling. In a continuous miniplant, the process was operated for over 90 h, with a period of stable operation for 60 h during which an average amine yield of 61% was achieved. Water accumulation was found to correlate with the decreasing yield over time, alongside other potential factors such as catalyst leaching and deactivation. Riemer et al. [123] applied the similar green methanol-based TMS to a dedicated, high-pressure continuous reductive amination process. To directly address the challenge of water accumulation (a coproduct), they innovatively integrated an organic solvent nanofiltration membrane for continuous water removal, maintaining its concentration below 3.1 wt%. The optimized continuous process achieved stable operation for over 90 h with constant amine yields >90% and low catalyst loss (0.003% Rh per hour to the product phase).

In addition, the applied magnetic field provides an effective strategy for catalyst immobilization, separation, and recovery in continuous-flow amination processes. By utilizing magnetic catalysts or magnetic nanoparticle supports, an externally applied magnetic field enables the efficient retention of the catalytic species within the reactor, enabling straightforward recycling by simply removing the field. Kim et al. [124] demonstrated a continuous, three-step reductive amination process converting nitroarenes and aldehydes to secondary amines using ammonia borane. One of their key innovations was the simple, reversible fixation of a Pd-Pt-Fe_3_O_4_ magnetic nanoflake catalyst inside tubular reactors using external neodymium magnets. This setup allowed the reaction to proceed at room temperature in good to excellent yields, and the catalyst could be easily recovered and reused by removing the magnets. In a biocatalytic approach, Božinović et al. [86] developed an efficient and sustainable process for furfurylamine synthesis. They covalently immobilized an ω-transaminase (N-His_6_-ATA-wt) onto functionalized magnetite nanoparticles. The biocatalyst was then introduced into a custom 3D-printed coil microreactor wrapped around a cylindrical permanent magnet, which held the particles in place. This magnetic field-assisted system enabled continuous operation for 18 days with a decrease in relative productivity for the enzymatic amination of furfural.

*Process Evaluation*

Process evaluation is a critical step in novel flow process development, as it provides a quantitative and holistic assessment of the environmental and economic viability of the new technology compared to established batch methods, guiding optimization and scale-up decisions. Yaseneva et al. [125] conducted a comprehensive assessment of a novel continuous-flow Buchwald–Hartwig amination process employing a Pd-NHC catalyst. The evaluation combined a detailed, cradle-to-gate Life Cycle Assessment (LCA) with a simplified economic analysis to compare it against a conventional batch process. The LCA revealed that while the synthesis of the novel Pd-NHC catalyst itself contributed significantly to certain environmental impact categories due to solvents like THF and DCM, the overall flow process demonstrated lower or comparable environmental scores for most indicators. This net benefit was attributed to the flow process enabling the use of a stoichiometric amount of the amine reagent and effectively reducing by-product formation. The study concluded that the continuous-flow process presents overall environmental advantages, providing a robust quantitative basis for the transition of such complex coupling reactions from batch to flow mode.

### 7.2. Kinetic Investigation

A fundamental understanding of reaction kinetics is paramount for process development, optimization, and scale-up of chemical transformations. However, elucidating accurate kinetic models for amination reactions, particularly complex networks like reductive amination, presents significant challenges in traditional batch reactors. The performance of the batch systems is often limited by factors such as mass and heat transfer, making it difficult to elucidate the intrinsic reaction kinetics. Continuous-flow reactors emerge as a powerful platform to overcome these obstacles and enable the collection of high-quality kinetic data, thus facilitating the development of reliable kinetic models. Consequently, employing flow chemistry for kinetic studies has become a valuable and effective approach in amination reactions.

Continuous-flow systems enable safe operation at elevated temperatures and pressures, providing an ideal platform of obtaining reliable kinetic data for nucleophilic substitution amination reactions [66,67,68,126]. Kim et al. [126] developed a comprehensive one-dimensional flow model incorporating mass/energy balances for nucleophilic aromatic substitution amination. By integrating the Reynolds number into the pre-exponential factor to account for mixing effects across different reactor diameters, the model enabled accurate kinetic parameter estimation and was subsequently applied for model-based design space identification and robustness analysis under pulse disturbances. Zhou et al. [66] employed a continuous-flow microreactor to safely conduct the high-temperature/pressure ammonolysis of 1-nitroanthraquinone, established a kinetics model based on the reaction mechanism to determine the apparent activation energy, and validated the reliability of extracting kinetic parameters under near-isothermal plug-flow conditions. Zhu et al. [67] leveraged a continuous-flow microreactor to safely study the high-temperature/pressure, non-catalytic amination of 4-chloronitrobenzene. A simplified kinetic model was established based on the mechanism to determine the apparent activation energy and pre-exponential factor, and the model’s predictive capability for conversion was validated under various conditions. Shen et al. [68] utilized a continuous-flow tubular reactor to study the apparent kinetics of the solvent- and catalyst-free aminolysis of ethyl acetate, revealing a zero-order reaction in the main conversion stage (20–80%) with a determined apparent activation energy, and suggested a potential autocatalytic effect of the product.

The application of continuous-flow reactors is especially advantageous for elucidating the complex reaction networks and determining the rate-limiting step in reductive amination [40,51]. Zhang et al. [40] established a detailed kinetic model for the complex, consecutive reductive amination network of benzaldehyde to benzylamine with the Ni/SiO_2_ catalyst in a micropacked-bed reactor. Their study determined the reaction rate constants and activation energies for the key steps and revealed that the rate-determining step may switch between imine condensation and hydrogenation depending on ammonia concentration. In addition, the same group [51] also established a mechanistically grounded kinetic model focusing on the predominant route from benzylimine to dibenzylamine catalyzed by Pt/SiO_2_, involving multiple parallel and consecutive pathways. The key kinetic insight revealed that the hydrogenation rate of the dibenzylimine intermediate was significantly faster than that of benzylimine, explaining the maintained low concentration of this key intermediate and the resulting high selectivity towards the secondary amine by suppressing its side reaction with ammonia.

Slug flow platforms with integrated process analytics allow for rapid and material-efficient kinetic parameterization of homogeneous catalytic cycles. Wagner et al. [127] performed a kinetic investigation of Buchwald–Hartwig amination within an automated slug-flow platform by generating time-course data at multiple residence times, which was used to parameterize a mechanistic kinetic model based on a catalytic cycle. The model identified the oxidative addition step as rate-limiting and included a fitted side-reaction pathway, enabling accurate in silico performance prediction across the design space. Microreactors offer a safe and intensified environment for studying the kinetics of electrophilic amination reactions. Tan et al. [114] established a kinetic model for the N-amination of 3,5-diamino-4-nitropyrazole in a continuous-flow microreactor, determining that the reaction follows first-order kinetics based on the in situ generation of the NH_2_^+^ electrophile, and obtained the corresponding rate constants across a temperature range of 50–70 °C, showing excellent agreement with the experimental data.

Kinetic investigation of enzymatic amination in continuous packed-bed reactors reveals complex interactions and provides essential insights for the rational engineering of immobilized multi-enzyme systems [89,90]. Franklin et al. [89] demonstrated that the reaction kinetics in a packed-bed flow reactor with co-immobilized amine dehydrogenase and formate dehydrogenase significantly deviated from classical Michaelis–Menten behavior. The primary reasons were a high enzyme concentration relative to the cofactor NADH, leading to underutilization of the catalyst and competitive product inhibition by the chiral amine. The study developed an improved rate law accounting for these factors, which was crucial for accurately interpreting the limited conversion and for guiding future reactor and process design. Finnigan et al. [90] synergistically combined mechanistic modeling (based on Michaelis–Menten kinetics) and empirical modeling (via a definitive screening design) to rapidly optimize a co-immobilized two-enzyme flow system for reductive amination. The initial mechanistic model provided a crucial starting point for the empirical model, though its predictive limits were reached under intensified flow conditions where enzyme concentration exceeded substrate levels. Subsequently, the developed empirical model successfully captured the complex interactions within the design space, enabling the model-based derivation of two distinct optimized processes.

From a detailed analysis of the flow amination cases examined in this work, cases involving reductive amination account for over 40%, while other reaction types such as nucleophilic amination, electrophilic amination, and catalytic coupling amination also constitute a significant proportion. Packed-bed and tubular reactors are predominantly used, with the former employed in approximately 50% of the cases and the latter in over 30%. Catalysts are required in more than 70% of the continuous-flow amination processes, a substantial portion of which are solid catalysts. This directly explains why packed-bed reactors are so prevalent in continuous-flow amination processes. Over 30% of the cases provide an explicit comparison between batch and continuous-flow processes. The results demonstrate clear advantages of the continuous-flow process relative to batch operation. Although partial cases report that reaction outcomes such as yield are lower than in batch processes, the significant reduction in reaction time and substantial improvement in overall efficiency highlight the considerable value of flow chemistry technology in amination processes. Nevertheless, the application of flow chemistry in amination processes remains relatively underexplored. Many existing batch amination processes possess potential for enhancement through flow chemistry technology, yet related investigations are still limited. While this technology offers distinct advantages for studying reaction kinetics, research on the kinetics of amination reactions involving complex mechanisms remains insufficient. Furthermore, discussions concerning the scale-up of continuous-flow amination processes are relatively scarce, indicating a need for more in-depth research and practical development in these areas.

## 8. Conclusions

Flow chemistry, leveraging its unique advantages, has demonstrated significant promise in amination reactions. Over the past decade, a substantial number of flow-based amination cases have been reported. This review systematically examines the benefits offered by flow chemistry from a process intensification perspective across various scenarios such as heterogeneous amination, thermally activated amination, enzymatic amination, and photo-/electricity-driven amination. It also discusses the development and application of novel flow amination processes from perspectives like solid handling and catalyst separation, while highlighting the distinct advantages of flow chemistry in studying amination reaction kinetics. Despite notable progress for the continuous-flow amination reactions, several challenges remain to be addressed. For instance, the performance of many efficient catalysts of amination reactions originally developed for batch processes has not been sufficiently explored under continuous-flow conditions. There is still a lack of standardized scale-up guidelines from laboratory-scale flow setups to industrial continuous production for continuous-flow amination reactions. Furthermore, the complex mechanisms underlying many catalytic amination reactions have hindered in-depth kinetic studies, and the initial investment costs for flow systems tend to be higher compared to conventional batch processes. The combined progress in flow chemistry technology and the commercialization of its equipment has rendered this methodology increasingly accessible and cost-effective for implementation in academic and industrial research settings. By fostering deeper collaboration across chemistry and engineering disciplines, flow-based amination is positioned to assume a pivotal and expanding role in enabling sustainable, efficient routes to amine compounds.

## Figures and Tables

**Table 1:** Typical examples for continuous-flow solid–liquid amination processes ^a^.

Entry	Feed	Product	Catalyst	Parameter	Result
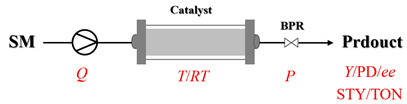					
1 [23]	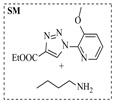	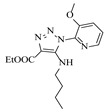	Cu(OAc)_2_·H_2_O& K_3_PO_4_	*T* 80 °C *RT* 30 min *P--* Toluene	*Y* 92% PD 0.368 mmol/h STY 0.123 mmol/(h mL)
2 [24]	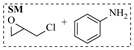	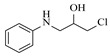	MoO_3_/TiO_2_-ZrO_2_-1C	*T* r.t. *Q* 0.2 mL/min *P--* EtOH	*Y* ~ 80% PD 4.9 mmol/h
3 [25]	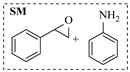	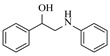	ZrOTf-BTC	*T* r.t. *Q* 1 mL/min *P--* CH_2_Cl_2_	*Y* 83–93% TON > 2700 (30 h)
4 [26]	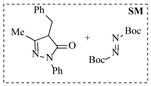	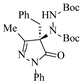	LP-IV	*T* r.t. *RT* 10 min *P--* DCM/PhMe	*Y* 86% PD 3.05 mmol·mmolcat^−1^·h^−1^
5 [27]	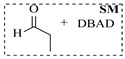	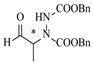	H-Pro-Asp-NH-TentaGel	*T* r.t. *Q* 0.1 mL/min *P* 60 bar CHCl_3_	*Y* 83–91% 88–92% ee (20 h)
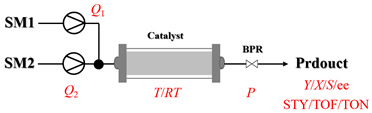					
6 [28]	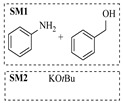	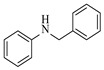	Fe_SA_@N-G	*T* 150 °C *Q*_1_ 0.013 mL/min *Q*_2_ 0.012 mL/min *P* 10 bar Toluene	*Y* 90%
7 [29]	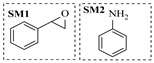	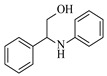	MgO-SiO_2_-IL	*T* 60 °C *RT* 120 min *P* 2 bar Toluene	*X* 81.1~99.4% *S* 95.9~98.6% TOF 38.4~48.8 h^−1^ (72 h)
8 [30]	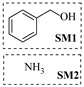	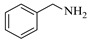	36 wt% Ni -Al_2_O_3_/SiO_2_	*T* 160 °C *RT* 46.7 min *P* 60 bar Oxylene	*X* 100% *S* 100% TOF 2.5 × 10^−4^ min^−1^
9 [31]	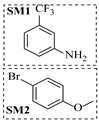	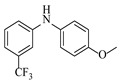	silica-grafted Pd−PEPPSI−IPentCl	*T* 85 °C *RT* 17 min *P--* DME/THF	*Y* 86%
10 [32]	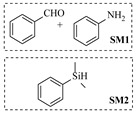	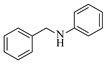	Au@APTES@SBA	*T* r.t. *RT* 18 min *P--* IPA	*Y* 85% TON 70
11 [33]	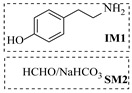	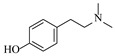	-- (pic-BH_3_ served as a reducing agent)	*T* r.t. *Q*_1_ 0.54 mL/min *Q*_2_ 0.1 mL/min *P--* MeCN/H_2_O	*Y* 77% STY 11.4 g/(L·h)
12 ^b^ [34]	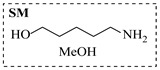	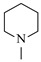	γ-Al_2_O_3_	*T* 340 °C *Q*_1_ 0.5 mL/min *Q*_2_ 0.3 mL/min *P* 100 bar MeOH/scCO_2_	*Y* 94%
13 ^c^ [35]	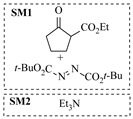	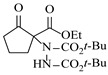	PS-TU	*T* r.t. *RT* 21 min *P--* Toluene	*X* 82~99% 91~94% ee (7.5 h)
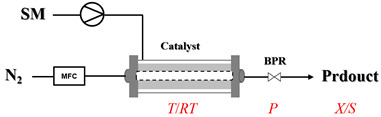					
14 [36]	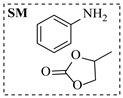	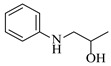	Br/APS/PAIHF trifunctional organocatalyst	*T* 140 °C *RT* ~ 5 min *P* Amb. pressure *--*	*X* 61% *S* 97.5%

^a^ Abbreviations in the table: *Q*—flow rate, *T*—temperature, *RT*—residence time, *P*—pressure, *X*—conversion, *Y*—yield, *S*—selectivity, PD—productivity, ee—enantiomeric excess, STY—space time yield, TON—turnover number, TOF—turnover frequency. ^b^ Supercritical CO_2_ served as the solvent. ^c^ The SM2 stream, introduced via a switching valve, serves to periodically regenerate the deactivated catalyst by neutralizing the protonated basic sites.

**Table 2:** Typical examples for gas–liquid–solid flow amination processes catalyzed by earth-abundant metal catalysts ^a^.

Entry	Substrate	Nitrogen Source	Product	Catalyst	Parameter	Result
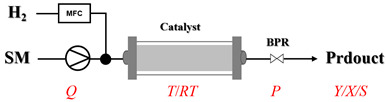						
1 [37]	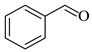	NH_3_	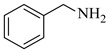	Co@Cs	*T* 80 °C *Q* 0.5 mL/min *P* 1.0 MPa MeOH	*X* > 99% *S* > 99%
2 [38]	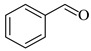	NH_3_	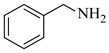	Ni_1_Al_2_-Cs_1_._0_	*T* 50 °C *RT* 3.5 min *P* 1.0 MPa MeOH	*Y* 98~99% (>500 h)
3 [39]	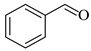	NH_3_	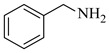	Fe_2_P NC/ZrO_2_	*T* 200 °C *Q* 2 mL/min *P* 4 MPa EtOH	*Y* > 80%(30 h)
4 [40]	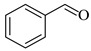	NH_3_	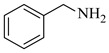	Ni/SiO_2_	*T* 70 °C *RT* 3.5 min *P* 1.0 MPa MeOH	*Y* 99%
5 [41]	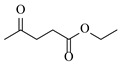	C_6_H_13_NH_2_	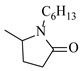	Ni_2_P/SiO_2__A600	*T* 170 °C *Q* 0.33 mL/min *P* 10 bar Toluene	*X* > 99.5% *Y* 98%
6 [42]	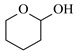	NH_3_	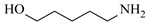	10Ni-HAP	*T* 60 °C WHSV_2-HTHP_ 0.5 h^−1^ *P* 2 MPa H_2_O	*X* 100% *S* 67~74% (80 h)
7 [43]	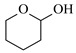	NH_3_	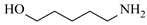	Ni/ZrO_2_	*T* 80 °C WHSV_2-HTHP_ 0.5 h^−1^ *P* 3 MPa H_2_O	*X* 100% *S* ~83–~67% (90 h)
8 ^b^ [44]	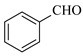	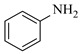	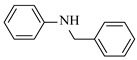	CuAlO_x_	*T* 120 °C *Q* 0.5 mL/min *P* 2 MPa MeOH	*Y* > 95% (6 h)
9 [45]	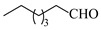	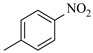	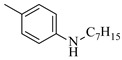	Cu/Al_2_O_3_	*T* 115 °C *Q* 0.35 mL/min *P* 50 bar Toluene	*Y* > 80% (2 h)
10 [46]	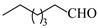	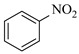	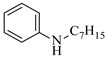	Ag/Al_2_O_3_	*T* 100 °C *Q* 0.5 mL/min *P* 3.0 MPa Toluene	*Y* > 48% (2.5 h)

^a^ Abbreviations in the table: *Q*—flow rate, *T*—temperature, *RT*—residence time, *P*—pressure, *X*—conversion, *Y*—yield, *S*—selectivity. ^b^ Condensation of primary amines with aromatic aldehydes was performed in a batch reactor.

**Table 3:** Typical examples for continuous-flow gas–liquid–solid flow amination processes catalyzed by noble metal catalysts ^a^.

Entry	Feed	Product	Catalyst	Parameter	Result
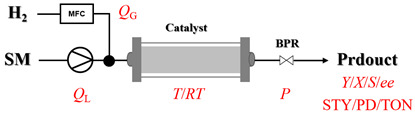					
1 [47]	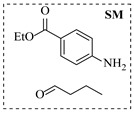	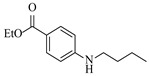	ESi-Pd	*T* 50 °C *Q*_L_ 0.3 mL/min *Q*_G_ 9.5 mL/min *P--* THF/EtOH	*Y* 85% STY 3.59 kg/(L·day)
2 [48]	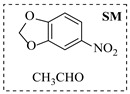	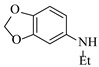	Pt/C (5 wt%)	*T* 40 °C *RT* 1.4 min *P* 0.7 MPa CH_3_CN	*Y* 87% PD 0.26 g/h
3 [49]	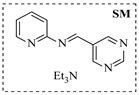	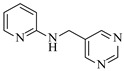	5 wt% Pd(OH)_2_/C	*T* 70 °C *RT* 6.5 min *P* 3.0 MPa MeOH/Toluene	*Y* 85~90% STY(Toluene) 16.84 g/(L·h) STY(MeOH) 17.64 g/(L·h)
4 [50]	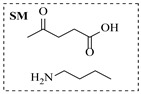	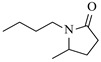	Ru-HC	*T* 80 °C *Q*_L_ 0.1 mL/min *P* 40 bar MeOH	*X* 99% *S* 98%
5 ^b^ [51]	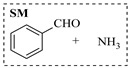	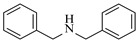	Pt/SiO_2_	*T* 30 °C *RT* 3.5 min *P* 2 MPa MeOH	*Y* 95.5%
6 [52]	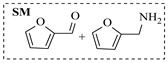	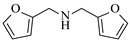	Pd-N/Ca	*T* 25 °C *Q*_L_ 0.3 mL/min *P* 30 bar MeOH	*X* 100% *S* 89%
7 [53]	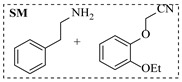	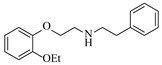	DMPSi-Pd/ AC-CP(3:1)	*T* 80 °C *Q*_L_ 0.1 mL/min *Q*_G_ 20 mL/min *P--* Toluene/EtOH	*Y* 91–96%
8 [54]	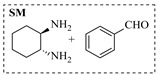	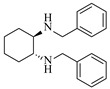	5% Pt/C	*T* 40 °C *Q*_L_ 0.2 mL/min *Q*_G_ 20 mL/min *P--* Toluene/EtOH	*--*
9 [55]	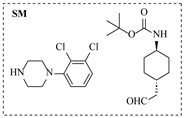	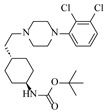	5% Pt/C	*T* 80 °C *Q*_L_ 0.5 mL/min *P* Atm. pressure Toluene/MeOH	*X* 100% *S* 97%
10 [56]	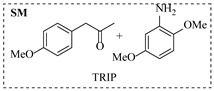	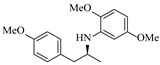	PS-Ir D	*T* r.t. *Q*_L_ 0.07~0.08 mL/min *Q*_G_ 5.0 mL/min *P* 0.2 MPa Toluene	*Y* 81–95% 91–92% ee TON ~ 100
11 [57]	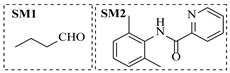	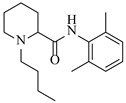	10% Pd/C	*T* 55 °C *Q*_L_ 0.5 mL/min *P* 30 bar MeOH/AcOH	*Y* 89%
12 [58]	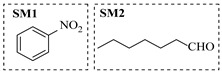	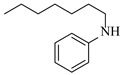	Au/Al_2_O_3_	*T* 80 °C *Q*_L_ 0.5 mL/min *Q*_G_ 60 mL/min *P* 50 bar Toluene	*Y* 90~95% (135 min)
13 [59]	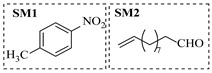	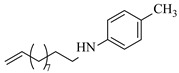	Au/Al_2_O_3_	*T* 90 °C *Q*_L_ 0.5 mL/min *Q*_G_ 60 mL/min *P* 50 bar Toluene	*Y* 88~96% (154 min)
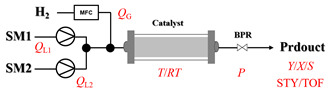					
14 [60]	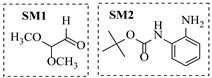	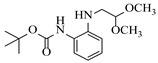	10% Pd/C	*T* 100 °C *RT* ~ 8 s *P* 10 bar *i*PrOAc	*X* > 99% *S* > 99%
15 [61]	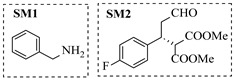	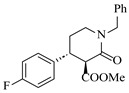	5% Pt/C	*T* 100 °C *Q*_L1_ 0.1 mL/min *Q*_L2_ 0.1 mL/min *Q*_G_ 15 mL/min *P* 3 bar 2-MeTHF	*X* 100% *S* 100% 93:7(trans/cis)
16 [62]	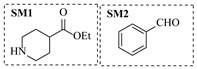	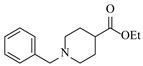	Pt/C	*T* 80 °C *Q*_L1_ 0.1 mL/min *Q*_L2_ 0.1 mL/min *Q*_G_ 15 mL/min *P--* Toluene	*Y* 100% TOF 24 h^−1^ STY 3.9 kg/(L·day)
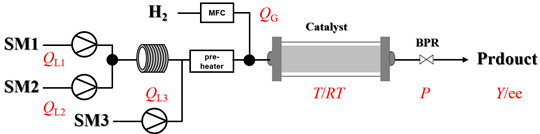					
17 [63]	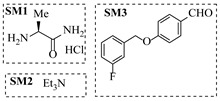	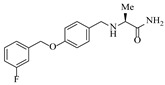	3% Pt/C−S	*T* 110 °C *Q*_L1_ 0.1 mL/min *Q*_L2_ 0.1 mL/min *Q*_L3_ 0.1 mL/min *Q*_G_ 5 mL/min *P* 0.4 MPa MeOH/4-MeTHP	*Y* 96% >99% ee
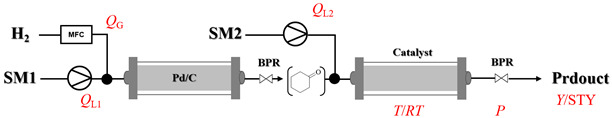					
18 [64]	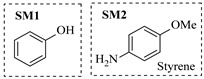	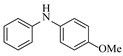	Pd(OH)_2_/C	*T* 140 °C *Q*_L1_ 0.1 mL/min *Q*_L2_ 0.1 mL/min *Q*_G_ 5.0 mL/min *P* 0.5 MPa Toluene	*Y* 96% STY 0.31 kg/(L·day)

^a^ Abbreviations in the table: *Q*—flow rate, *T*—temperature, *RT*—residence time, *P*—pressure, *X*—conversion, *Y*—yield, *S*—selectivity, ee—enantiomeric excess, STY—space time yield, PD—productivity, TON—turnover number, TOF—turnover frequency. ^b^ Residence time of liquid in micropacked-bed reactor.

**Table 4:** Typical examples for continuous-flow amination via thermal activation without catalysts ^a^.

Entry	Feed	Product	Parameter	Result
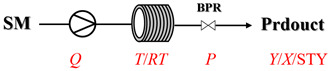				
1 ^b^ [65]	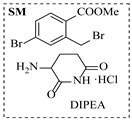	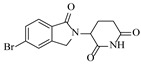	*T* 220 °C *RT* 6 min *P* 5 MPa MeCN	*Y* 71% STY 53 g/h
2 [66]	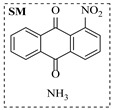	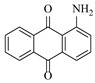	*T* 213 °C *RT* 4.3 min *P* 1000 psi NMP	*X* 98.4% *Y* ~ 88%
3 [67]	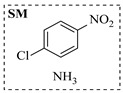	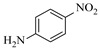	*T* 234 °C *RT* 75 min *P* 750 psi NMP	*X* 98.8% *Y* ~ 91%
4 [68]	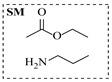	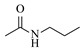	*T* 218 °C *RT* 350 min *P* 3.5 MPa None	*X* 94.8%
5 ^c^ [69]	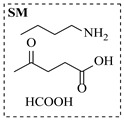	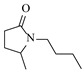	*T* 140 °C *RT* 10 min *P--* MeCN	*X* 93% *Y* 86%
6 [70]	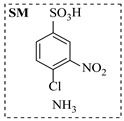	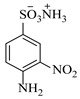	*T* 190 °C *RT* 30 min *P* 28 bar H_2_O	*X* 100% *Y* 96%
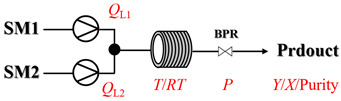				
7 [71]	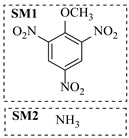	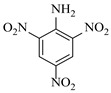	*T* 70 °C *RT* 0.5 min *P* 100 psi THF	*X* 100% Purity 100%
8 [72]	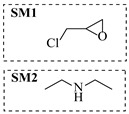	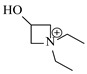	*T* 80 °C *RT* 60 min *P* 10 bar EtOH	*Y* 66%
9 [73]	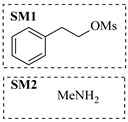	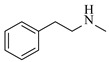	*T* 140 °C *RT* 4 min *P* 300 psi MeOH/H_2_O	*Y* 90%
10 [74]	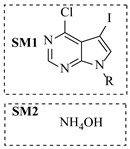	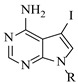	*T* 130 °C *RT* 60 min *P* ~ 450 psi Dioxane/ethanol	*Y* 90.1%
11 [75]	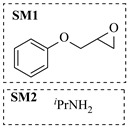	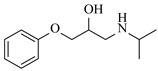	*T* 120 °C *RT* 20 min *P* 5.2 bar Toluene/EtOH	*X* 100%
12 [76]	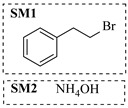	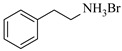	*T* 160 °C *RT* 10 min *P* 350 psi MeOH/H_2_O	*Y* 94%
13 [77]	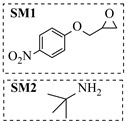	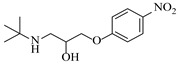	*T* 120 °C *RT* 20 min *P* 6 bar Toluene/EtOH	*X >* 99% *Y* 66%
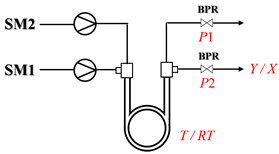				
14 [78]	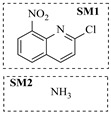	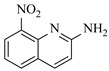	*T* 150 °C *RT* 30 min *P*1 350 psi *P*2 300 psi DMSO/H_2_O	*X* 95% *Y* 93%
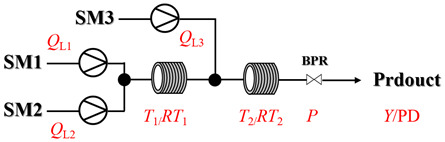				
15 [79]	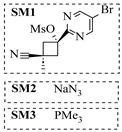	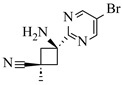	*T*_1_ 150 °C *T*_2_ 60 °C *RT*_1_ 5.9 min *RT*_2_ 2.45 min *P* 10 bar DMSO/H_2_O-Toluene	*Y* 59% PD 12 g/h

^a^ Abbreviations in the table: *Q*—flow rate, *T*—temperature, *RT*—residence time, *P*—pressure, *X*—conversion, *Y*—yield, STY—space time yield, PD—productivity. ^b^ The result here is the reaction outcome of the telescoped continuous process incorporating the amination step. ^c^ A glass chip microreactor was adopted in this example.

**Table 5:** Typical examples for thermally activated and catalytic amination processes operated in a continuous-flow system ^a^.

Entry	Feed	Product	Catalyst	Parameter	Result
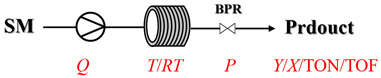					
1 [80]	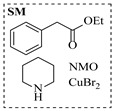	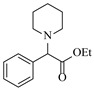	CuBr_2_	*T* 100 °C *RT* 25 min *P--* DMSO	*Y* 83% TON 49.8
2 [81]	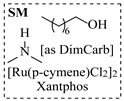	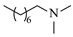	[Ru(*p*-cymene)Cl_2_]_2_ Xantphos	*T* 290 °C *RT* 15 min *P* 70 bar *t*-amyl alcohol	*X* 97% TOF 776 h^−1^
3 [82]	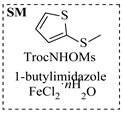	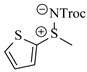	FeCl_2_·*n*H_2_O	*T* 80 °C *RT* 1 min *P* 100 psi EtOAc	*Y* 95%
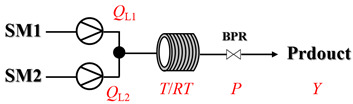					
4 [83]	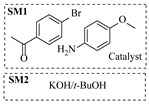	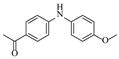	*t*-BuXPhosPd(π-cinnamyl)OTf	*T* 100 °C *RT* 20 min *P* 4 bar *n*-PrOH/H_2_O	*Y* 89%
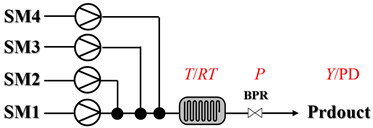					
5 [84]	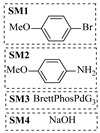	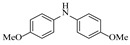	BrettPhosPdG_3_	*T* 100 °C *RT* 1.8 min *P* 100 psi 2-MeTHF/THF/MeOH	*Y* 72% PD 436 mg/h
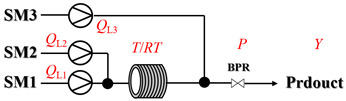					
6 [85]	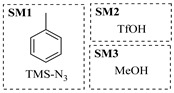	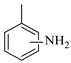	TfOH	*T* 90 °C *RT* 2.9 min *P* 7 bar CHCl_3_	*Y* 78%

^a^ Abbreviations in the table: *Q*—flow rate, *T*—temperature, *RT*—residence time, *P*—pressure, *X*—conversion, *Y*—yield, PD—productivity, TON—turnover number, TOF—turnover frequency.

**Table 6:** Typical examples for continuous-flow enzymatic amination processes ^a^.

Entry	Feed	Product	Catalyst	Parameter	Result
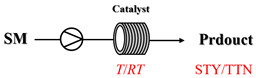					
1 [86]	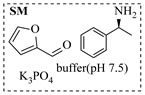	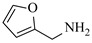	*N*-His_6_-ATA-wt	*T* r.t. *RT* 44 min	STY_max_ 1.07 g/(L·h) TTN 2.04 × 10^7^(18 d)
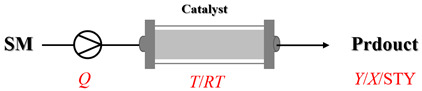					
2 [87]	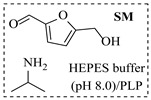	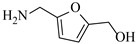	ATA-Spo	*T* 21 °C *RT* ~ 4 min	*X* 41~75% (12 days)
3 [88]	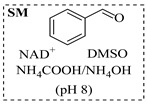	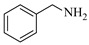	LE-AmDH-v1/ Cb-FDH	*T* 40 °C *Q* 0.02 mL/min	*Y* 47% STY 7.4 g/(L·day)
4 [89]	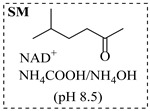	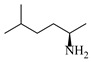	AmDH/FDH	*T* 40 °C *RT* 11.9 min	*X* 48%
5 ^b^ [90]	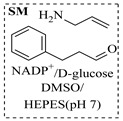	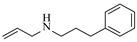	*Ad*RedAm/ *Bs*GDH	*T* 30 °C *RT* 10 min	*X* 98% *Y* 44% STY 10.3 g/(L·h) (132 h)
6 ^c^ [91]	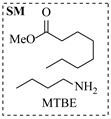	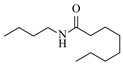	CAL-B	*T* 50 °C *RT* 10 min	*X* 98%
7 [92]	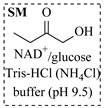	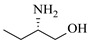	AmDH wh84/GDH	*T* 30 °C *Q* 0.2 mL/min	*X* 91.8% (48 h)
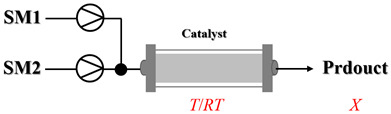					
8 [93]	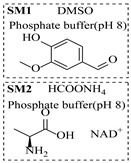	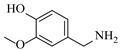	HeWT/HeAlaDH + CbFDH	*T* 25 °C *RT* 20 min	*X* 40%
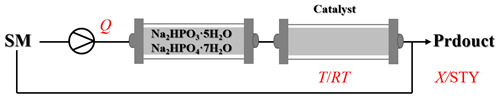					
9 [94]	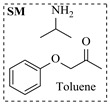	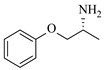	AsR-wTA	*Q* 0.2 mL/min *T* 25 °C *RT* 9.1 min	*X* ~ 70% STY 1.99 g/(L·h) (72 h)
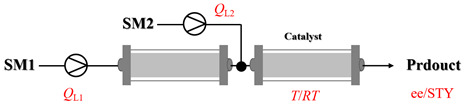					
10 [95]	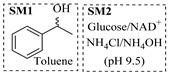	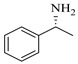	*Ja*AmDH&GDH @DON	*T* 30 °C *Q*_L1_ 0.02 mL/min *Q*_L2_ 0.09 mL/min	*Y* 97% 99% ee
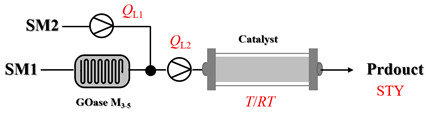					
11 [96]	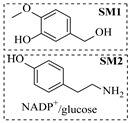	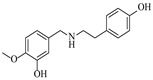	IR80-GDH	*T --* *Q*_L1_ 0.02 mL/min *Q*_L2_ 0.02 mL/min	STY 2.26 g/(L·h)
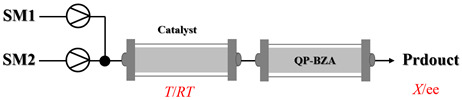					
12 [97]	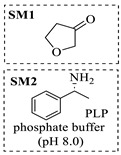	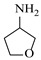	*He*WT	*T* 37 °C *RT* 5 min	*X* 98% 30% ee (*S*)
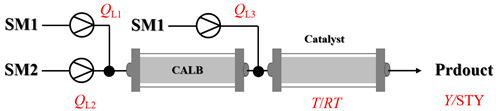					
13 [98]	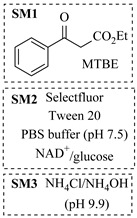	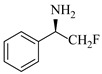	AmDH&GDH@DON	*T* 30 °C *Q*_L1_ 0.08 mL/min *Q*_L2_ 0.008 mL/min *Q*_L3_ 0.024 mL/min	*Y* 85~98% STY 17.1~19.7 g/(L·h) (96 h)
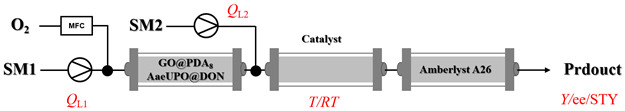					
14 [99]	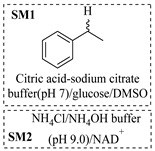	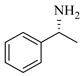	*Cps*ADH-*Ja*AmDH	*T* 30 °C *Q*_L1_ 0.03 mL/min *Q*_L2_ 0.03 mL/min	*Y* 96% 99% ee STY 3.2 g/(L·h)
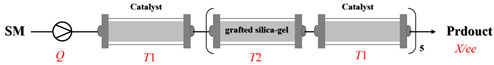					
15 [100]	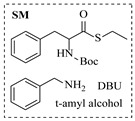	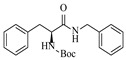	Alcalase	*T*1 50 °C *T*2 150 °C *Q* 0.2 mL/min	*X* 79% 98% ee

^a^ Abbreviations in the table: *Q*—flow rate, *T*—temperature, *RT*—residence time, *X*—conversion, *Y*—yield, ee—enantiomeric excess, STY—space time yield. ^b^ The continuous-flow process was operated at 75 psi. ^c^ The continuous-flow process was operated at 100 psi.

## Data Availability

No new data were created or analyzed in this study. Data sharing is not applicable to this article.
